# Effects of unilateral single-mode balance training compared to combined balance and plyometric training on soccer players’ interlimb asymmetry in static and dynamic balance performance

**DOI:** 10.1371/journal.pone.0358565

**Published:** 2026-09-16

**Authors:** Thomas Muehlbauer, Reinhold Kliegl, Katharina Borgmann, Sam Limpach, Dirk Krombholz, Stefan Panzer

**Affiliations:** 1 Division of Movement and Training Sciences/Biomechanics of Sport, University of Duisburg-Essen, Essen, Germany; 2 Department of Sport and Health Sciences, University of Potsdam, Potsdam, Germany; 3 Institute of Biomechanics and Orthopaedics, German Sport University Cologne, Cologne, Germany; 4 Institute of Sport Science, Saarland University, Saarbrücken, Germany; 5 Dynamo Dresden, Dresden, Germany; 6 Department of Health and Kinesiology, Texas A&M University, College Station, Texas, United States of America; Martin-Luther-University Halle-Wittenberg, GERMANY

## Abstract

**Background:**

The effects of training interventions on interlimb balance performance have been recently investigated. However, no comparison was conducted between the impact of a single-mode versus combined training modality, whereby complementary adaptations are suggested for the latter.

**Objective:**

Thus, we investigated the effects of a single-mode versus combined training modality on interlimb static and dynamic balance performance in soccer players.

**Methods:**

Young male soccer players (*N* = 57, Tier level 3) were randomly allocated to a unilateral single-mode balance training (BT) group (*n* = 17, age: 13.8 ± 1.5 years), a unilateral combined balance and plyometric jump training (BT + PT) group (*n* = 20, age: 14.1 ± 1.5 years) or an active control (CON) group (*n* = 20, age: 14.2 ± 1.6 years). Training was conducted in-season for nine weeks (two sessions per week) using the non-dominant leg. Pre- and post-intervention, unipedal balance performance was assessed for both legs under static (unipedal stance only) and dynamic (unipedal stance with continuous leg swing) task conditions while increasing the difficulty level (i.e., gradual reduction of the base of support). Binary (success rate) and metric (center of pressure [CoP] indices, limb symmetry index [LSI]) parameters of postural control were calculated.

**Results:**

Both training modalities yielded significant improvements in interlimb static and dynamic postural control, with greater effects in the single-mode BT group for the parameter success rate and in the combined BT + PT group for the CoP-based indices. Significant improvements in the LSI occurred irrespective of the training modality.

**Conclusion:**

Both, single-mode BT (binary outcome) and combined BT + PT (metric outcome) appear to be effective in improving interlimb static and dynamic postural control in young male soccer players. Due to the floor and ceiling effects across outcome domains, it cannot be concluded that one training mode is superior to the other. However, the more in-depth analysis (i.e., CoP-based indices) yields findings in favor of the combined BT + PT and suggests that regulatory processes, in particular, benefit from this training modality.

## Introduction

Research indicates that substantial differences between the lower limbs in postural control are linked to an elevated risk for sustaining a time-loss lower-extremity injury [1,2]. Specifically, Smith et al. [2] evaluated balance performance in collegiate athletes from different sports (e.g., soccer) and tracked lower-limb injuries over the course of a season. Of the 184 participants, 81 sustained non-contact injuries. The authors also found that athletes with a greater than 4 cm difference in anterior reach between the left and right legs had a 2.2-fold higher injury risk. There is further evidence from a performance-related perspective showing that interlimb asymmetry in postural control is related to competition level in soccer players [3] which could result in worse athletic performance [4]. Precisely, Gonzalez-Fernandez et al. [3] reported that greater interlimb asymmetry in balance performance depends on the competition level, with the largest values observed among amateur soccer players (5.3–12.0%), followed by semi-professional players (4.0–9.0%), and professional players (1.7–4.3%). For athletes including soccer players, interlimb asymmetry values greater than 10% are considered maladaptive and clinically meaningful [5–7]. Furthermore, there is evidence that asymmetry increases following fatigue [8], which can certainly occur in soccer due to long or intense periods of training/competition. Consequently, training interventions capable of reducing interlimb asymmetry in postural control are needed to mitigate injury risk and limit declines in athletic performance.

Several studies [9–11] have examined how unilateral single-mode balance training (BT) affects balance performance in both the trained and untrained lower extremity. Rasool and George [9] investigated athletes who completed four weeks of unilateral BT. For the training but not for the control group, they observed significant improvements in balance performance in the trained limb and, to a lesser extent, in the untrained limb. Similarly, Oliveira et al. [10] reported that adults who undertook six weeks of unilateral BT showed significant enhancements in postural sway in the trained limb, and smaller improvements in the untrained leg, relative to the control group. These results are encouraging, as they demonstrate both ipsilateral training effects on the practiced limb and contralateral transfer effects on the non-practiced limb. However, none of these studies compared pre- to post-intervention interlimb static and dynamic postural control using test conditions with a progressive increase in task difficulty. Therefore, it is unresolved whether unilateral BT is effective in reducing interlimb asymmetry in balance performance under less but also more difficult test conditions as they occur in constantly changing training and game situations in soccer.

Further, the training protocols used in the aforementioned studies—as well as those summarized in recent reviews [12–14]—were limited to balance exercises, even though growing evidence [15–19] suggests that combining balance and plyometric (i.e., explosive movements focusing on fast and powerful contractions) exercises yields greater benefits than single-mode BT by leveraging specific but complementary adaptive mechanisms [20,21].

Accordingly, the purpose of the present study was to address the previously noted limitations and to examine the effects of nine weeks of unilateral single-mode BT compared with unilateral combined balance and plyometric jump training (BT + PT), implemented alongside regular soccer training, on interlimb asymmetry in static and dynamic postural control in young male soccer players. With respect to the results reported in the literature [9–11,13,16,18,19,22], we assumed that both single-mode BT and combined BT + PT would improve soccer players’ static and dynamic balance performance (i.e., increase in success rate, decrease in center of pressure [CoP] indices, increase in interlimb symmetry index). However, the effects would be greater for the latter training modality due to complementary adaptations caused by the combination of balancing and ballistic muscle power exercises.

## Materials and methods

### Participants

Fifty-seven young male soccer players participated in this study and were recruited (06/05/2024 to 26/07/2024) from the under 13 (11–12 years), under 15 (13–14 years), and under 17 (15–16 years) squads of a soccer club (i.e., SG Dynamo Dresden e. V.). All participants played at the highest or second highest league of their age (Tier level 3) [23]. The players were assigned to a unilateral single-mode BT group (*n* = 17, age: 13.8 ± 1.5 years; stature: 169.7 ± 10.9 cm; body mass: 58.8 ± 11.8 kg; maturity offset: 0.62 ± 1.19 years from peak height velocity [PHV]), a unilateral combined BT + PT group (*n* = 20, age: 14.1 ± 1.5 years; stature: 171.0 ± 12.4 cm; body mass: 58.5 ± 14.4 kg; maturity offset: 0.23 ± 1.40 years from PHV) or an active control (CON) group (*n* = 20, age: 14.2 ± 1.6 years; stature: 171.4 ± 11.9 cm; body mass: 57.3 ± 19.4 kg; maturity offset: 0.40 ± 1.36 years from PHV). To guard against potential confounding effects of between-subject differences in chronological age, maturity offset, and intervention type, we randomly assigned groups of 6–8 players per age group (i.e., U13, U15, U17 squads) to one of the three interventions. This cluster randomization was effective because there were no significant differences between groups in these two covariates. Consequently, they and their interactions with experimental factors were not included in statistical models to avoid overparameterization. All players were free of any musculoskeletal disorders, neurological impairments, or orthopedic issues within the previous three months. Ethical permission (approval number: TM_04.06.2020) was given by the Human Ethics Committee at the University of Duisburg-Essen, Faculty of Educational Sciences. Participants’ assent and parents’ written informed consent were obtained prior to the start of the study.

### Testing procedure

**Fig 1:**
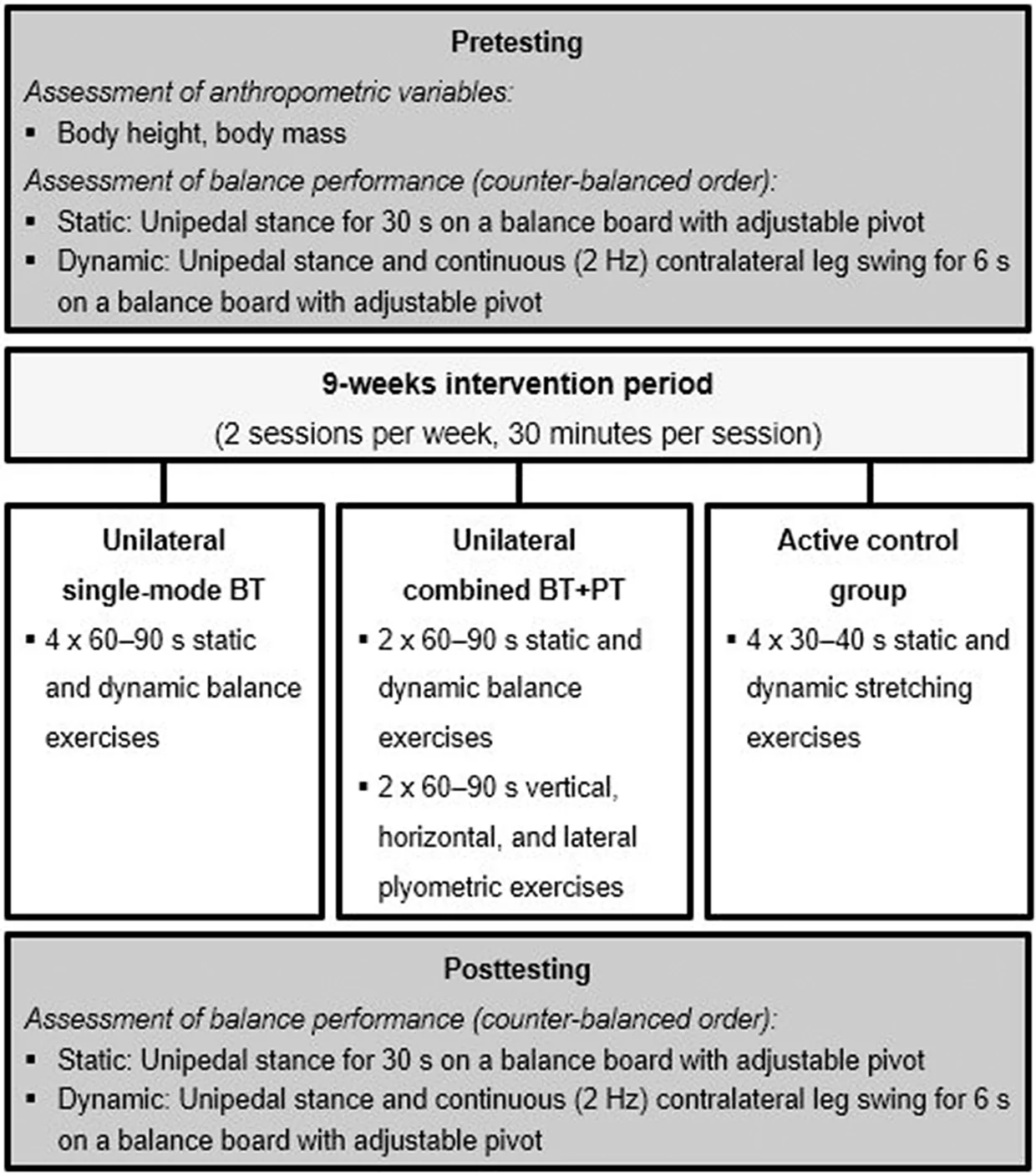
Flow chart illustrating the different study phases. *Note:* Unilateral exercises were conducted using the non-dominant leg (i.e., the stance leg during ball kicking). BT = unilateral single-mode balance training group; BT + PT = unilateral combined balance and plyometric jump training group.

The study design is illustrated in Fig 1. The pre- and post-intervention testing was administered in the team’s training facilities by the same experienced assessors (certified athletic coaches and graduated sport scientists). All players received standardized verbal instructions and a visual demonstration for each assessment, which included anthropometric measurements and static/dynamic balance testing. Before each testing session, every player completed a standardized 10-minute warm-up routine consisting of balance exercises and submaximal plyometric drills.

### Interventions

All experimental groups completed nine weeks of intervention (two sessions per week, 30 minutes for each session) during the competitive season, under the instruction and supervision of the club’s certified athletic coaches. Furthermore, it was ensured that training duration was matched between the groups. A detailed description of the interventions was provided by Muehlbauer and colleagues [24]. Briefly, the unilateral single-mode BT group performed static (e.g., upright stance) and dynamic (e.g., for-/back-/sideward leaning) balance exercise on balance boards, spinning tops, both sides utilized balls, and balance pads, executed while standing on the non-dominant leg (i.e., the stance leg during kicking). Each exercise session consisted of four sets of 60–90 s, interspersed with 30-s rest intervals. Training difficulty was progressively increased by extending exercise duration and modifying sensory input (i.e., change from the stance on firm/stable to the stance on foam/unstable surface) [25]. In the unilateral combined BT + PT group, each training session was divided into two components. The first component was similar to the single-mode BT described before but was reduced to two sets of 60–90 s. The second component comprised vertical, horizontal, and lateral plyometric exercises (e.g., box jumps, hurdle jumps, squat jumps) [26] with the non-dominant leg (used for take-off and landing). Training progression was achieved by gradually increasing the jump or hurdle height from 10 to 90 cm. The total number of ground contacts per session ranged from 108–162 in weeks 1–2, 90–168 in weeks 3–4, 114–166 in weeks 5–6, 108–162 in weeks 7–8, and 116–216 in week 9, aligning with values reported in previous studies involving young soccer players [27,28]. Participants in the active CON group performed both passive and active stretching exercises targeting the upper body (i.e., core, pectoral, and shoulder muscles) and lower body (i.e., calf, quadriceps, hamstring, and hip muscles). Each exercise consisted of four repetitions lasting 30–40 s, interspersed with 30-s rest intervals. Progression of the stretching program was implemented by increasing the duration of each exercise and transitioning from static to dynamic stretching movements. Chronic stretching leads to changes in the tension tolerance of muscles and connective tissue [29] but has only small to moderate effects on static and dynamic balance parameters [30]. All three groups also continued their regular training schedule, which included soccer-specific training (360–420 minutes per week), athletic training (90–180 minutes per week), and one competitive match each weekend. Soccer-specific sessions were held on match day + 2, −4, −3, −2, and −1, while athletic training sessions were conducted on match day + 2 and −3. In addition, players attended daily physical education classes from Monday to Friday between 08:00 and 10:45 a.m. Training load for all groups—including training frequency, duration, volume, intensity, and progression— was monitored by using standardized scoring sheets that documented relevant parameters (e.g., exercise duration, number of ground contacts). Training load was further regulated through weekly phone consultations between the study examiner and the certified athletic coaches.

### Assessments of anthropometric variables

Standing and sitting height were measured without shoes to the nearest 0.5 cm using a stadiometer (seca 217, Basel, Switzerland), and body mass was assessed in light clothing and barefoot to the nearest 100 g with an electronic scale (seca 803, Basel, Switzerland). Maturity offset was calculated as years from PHV using the regression equation provided by Mirwald et al. [31].

### Assessment of balance performance

**Fig 2:**
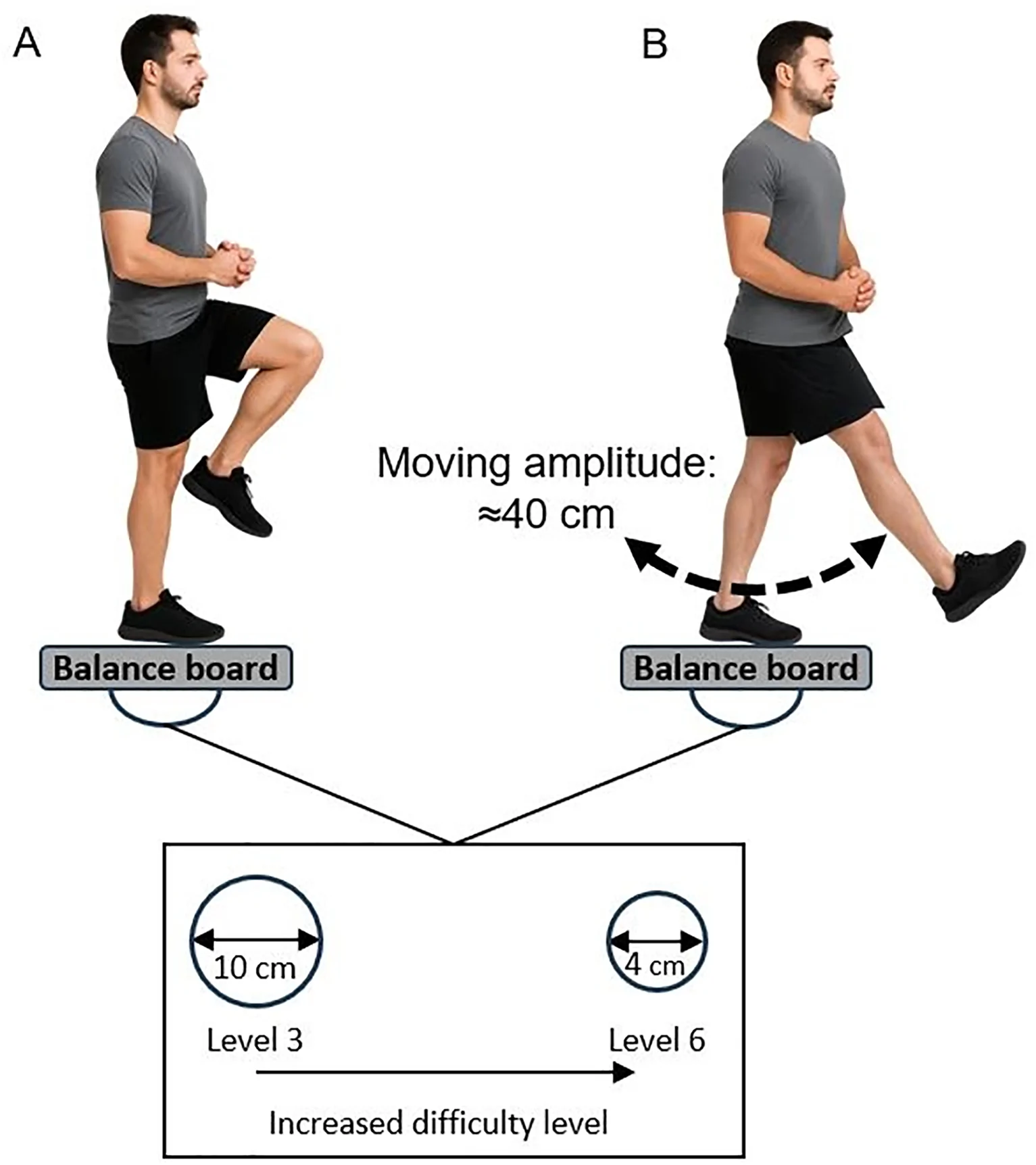
Schematic diagram for A) static (i.e., unipedal stance only) and B) dynamic (i.e., unipedal stance and continuous contralateral leg swing) balance assessment. *Note:* The balance board consists of an adjustable pivot to incrementally reduce the base of support diameter from 10 cm (level 3) to 4 cm (level 6). Participants completed the balance tasks with progressively increased difficulty level. The individual shown in this diagram was created by using ChatGPT (OpenAI, San Francisco, CA).

Balance performance was evaluated before and after the nine-week intervention period using a unipedal stance test. According to the conceptual framework of Paillard [32], the unipedal stance represents a contextualized, specific test condition—such as during kicking, crossing, and passing a soccer ball—related to the practiced type of sport (i.e., soccer) and is therefore suitable for an ecologically valid assessment of soccer players. Assessment was conducted for both the dominant and non-dominant leg, with limb dominance determined by self-report (“Which foot do you use to kick a ball?”) [33]. Players who reported using both sides were also asked which side they preferred — that is, which one they used more often. Participants stood upright with their hands on their hips and their gaze fixed on a board with an adjustable mechanical pivot (Wobblesmart©, Artzt GmbH, Dornburg, Germany) positioned on a force plate (Kistler; 9260AA, Winterthur, Switzerland) [34]. For the static balance test condition, participants were instructed to remain as still as possible for 30 s (Fig 2A) and in the dynamic balance test condition, they performed continuous leg swings with their contralateral leg and in accordance with a metronome set to 2 Hz for 6 s [35,36] (Fig 2B). The swing amplitude was standardized to 40 cm, marked by indicators positioned 20 cm in front of and 20 cm behind the participant [35]. Throughout the task, an assessor provided auditory feedback while visually monitoring the movement amplitude and frequency to ensure precise execution of the secondary motor task. Between-trial test-retest reliability data were calculated from the present study and yielded for the assessment of static balance ICC values (95% CI) of 0.50 (0.34, 0.65) for *ap*-COP RMS (dominant leg), 0.60 (0.46, 0.73) for *ap*-COP RMS (non-dominant leg), 0.55 (0.40, 0.68) for *ml*-COP RMS (dominant leg), and 0.60 (0.45, 0.72) for *ml*-COP RMS (non-dominant leg), indicating “moderate” reliability [37]. For dynamic balance, ICC values (95% CI) amounted to 0.50 (0.35, 0.65) for *ap*-COP RMS (dominant leg), 0.47 (0.31, 0.62) for *ap*-COP RMS (non-dominant leg), 0.41 (0.24, 0.58) for *ml*-COP RMS (dominant leg), and 0.52 (0.36, 0.66) for *ml*-COP RMS (non-dominant leg), indicating “poor to moderate” reliability [37].

In both tasks, difficulty was progressively increased by reducing the diameter of the support base on the adjustable pivot (ranging from level 3 [10 cm], indicating low difficulty, to level 6 [4 cm], indicating high difficulty). The rationale for evaluating static and dynamic balance across increasing difficulty levels was to account for individual differences in postural control, thereby minimizing potential floor effects (task too difficult) and ceiling effects (task too easy). Consequently, the task difficulty was adjusted incrementally for all players starting from level 3 over levels 4 and 5 to level 6. Using this procedure, the highest difficulty level that could be successfully achieved with both the dominant and non-dominant leg was determined for each player. For each difficulty level, two practice trials were performed to familiarize with the respective test condition followed by three data-collection trials, and the mean of the data-collection trials was used for further analyses. A trial was discarded and repeated if participants (a) lost their balance (e.g., touched the ground with the non-stance leg), (b) removed their hands from their hips, (c) failed to follow the metronome during the dynamic balance task, or (d) did not achieve the required movement amplitude during the dynamic balance task. The unipedal stance test is considered a valid measure of balance performance [38]. Compared to the step stance, the variance explained amounted to 58.5% (preferred leg) and 65.0% (non-preferred leg) for the static test condition and 63.2% (preferred leg) and 79.0% (non-preferred leg) for the dynamic test condition.

### Data analyses

Some of the data originates from a previously published study [24] in which the same interventions were used but different assessments were conducted. For each trial, two measures were computed from the CoP trajectories, i.e., the anteroposterior (*ap*) and mediolateral (*ml*) root mean square (RMS). Further, interlimb static and dynamic postural control was quantified with the limb symmetry index (LSI), following a formula by Bishop et al. [39]. Specifically, for each of the design cells, the *ap* and *ml* means of valid trials (max = 3) were computed and means of the dominant (D) and non-dominant (ND) leg were entered into the following equation:


$$\begin{array}{c} \text{LSI}\_\text{x }=\text{ min }(\text{x}\_\text{D},\text{ x}\_\text{ND})/\text{ max }(\text{x}\_\text{D},\text{ x}\_\text{ND})*\text{ 100 }[\%],\text{ where x }=ap\text{ or }ml \end{array}$$ (1)

An LSI value above 90% reflects interlimb symmetry in postural control, whereas values below this threshold indicate interlimb asymmetry.

For data analyses and graphics, we used mainly *tidyverse* (version 2.0.3) [40] and *easystats* (version 0.7.5.2*)* [41] packages in the R language (version 4.5.2) [42]*.* Generalized linear mixed model (GLMM) and linear mixed model (LMM) were estimated with *MixedModels*.jl (version 5.1.0) [43] in the Julia programming language (version 1.12.2) [44].

### Inferential statistics

We estimated fixed effects for the two contrasts defined for control and training groups, pretest/posttest, static/dynamic balance condition, task difficulty level, and dominant/non-dominant leg. The first contrast (C1) tested the average of the two training groups against the active CON group; the second contrast (C2) tested the difference between the two training groups. Contrasts with effect coding were used for the other factors. We also included all simple interactions between these main effects and six theoretically relevant three-factor interactions arising from the combination of time × (C1 | C2) × (balance condition | task difficulty level | leg). These contrasts and associated interactions were dictated by the design and specified *a priori* with a clear expectation about the profile of means, not as omnibus *F*-tests to be followed up with post-hoc *t*-tests adjusted for multiple comparisons [45,46]. This design was used both for the GLMM for the parameter success rate and the LMM for the CoP-based parameters. For unbalanced designs, (G)LMMs preserve statistical power better than repeated-measures ANOVAs. Box-Cox distribution analyses [47] revealed a pronounced non-normal data distribution for both *ap*-CoP and *ml*-CoP RMS values (S1 Fig). Therefore, a log-transformation was employed. We used log(*ap*) – log(*ml*) = log(*ap*/*ml*) as the primary dependent variable in the LMM for CoP-based parameters. Two additional LMMs with log(*ap*) and log(*ml*) as dependent variables were also planned to disambiguate results. Diagnostics of LMM residuals corroborated the log transformation of *ap*-CoP and *ml*-CoP RMS values (S2 Fig). Success of completing trials was analyzed with a GLMM with player as random factor. The full random-effect structure (RES) with variance components (VCs) and correlation parameters (CPs) for grand mean (GM), times, balance conditions, difficulty levels, and legs was supported by the data, VCs and CPs of the GLMM are documented in S1 Table (column “success”). There was no need for model selection [48]. The full RES for the log(*ap*/*ml*)- and log(*ap*)-LMMs was not supported by the data. Model pruning according to Bates et al. [48] using a 5-unit change in Akaike Information Criterion (AIC) as criterion led to a RES with VCs for GM, times, and balance conditions and zero-correlation parameters. The full RES provided the best goodness of fit for the log(*ml*)-LMM. VCs and CP of the three LMMs are documented in S1 Table (column “log [*ap/ml*]”, “log[*ap*]”, “log[ml]”).

While contrast specifications for all (G)LMMs were determined by the experimental design, the choice of measures was informed by the outcomes. Given respective ceiling and floor effects, the first decision was to analyze success rate only for two difficult conditions (levels 5 and 6) and CoP measures only for two easy conditions (levels 3 and 4). Aside from bypassing the interpretational problems, we reduced dependency with analyses of two non-overlapping subsets of the observations. As far as *ap*- and *ml*-CoP-measures were concerned, a distributional analysis clearly established the need for log-transformation to meet assumptions of normal distribution for model residuals. These are the primary dependent variables in this field, but for the present context, *ap*-CoP was expected to relate more directly to the theoretical issue of leg asymmetry (i.e., forward/backward sway). Thus, success rate and *ap*-CoP assessed with non-overlapping subsets of data were our primary choices. Further, we anticipated an interest in additional CoP analyses. Obviously, *ml*-CoP was to be checked as a complementary follow-up. Indeed, this analysis revealed some unexpected results that need to be followed up. Moreover, leg asymmetry is usually operationalized as a ratio between the two legs. Given that we analyzed log(CoP) values, the leg main effect was estimated as a ratio in the original metric. To align our analysis with the established practice of the field, we included a *post-hoc* LMM using this ratio directly as dependent variable. This analysis was carried out both for ratios of *ap*- and *ml*-CoP values. Finally, again to align our study with past practice, we also computed and analyzed leg asymmetry based on the established formula 1 for both *ap*- and *ml*-CoP.

## Results

### Preprocessing and overview

A total of 5,472 trials (i.e., 57 players × 2 times [pretest, posttest] × 2 legs [dominant, non-dominant] × 2 balance conditions [static, dynamic] × 4 task difficulty levels [3–6] × 3 trials [1–3]) were available. We carried out a preliminary analysis that determined constraints for inferential statistics. First, across the four levels of task difficulty, success was .98 for levels 3 and 4, but only .68 for levels 5 and 6. Therefore, we restricted the application of the GLMM for the parameter success rate to 2,736 trials resulting from levels 5 and 6 (i.e., avoiding the ceiling effects of levels 3 and 4) and the application of the LMMs for the CoP-based parameters to 2,668 trials from levels 3 and 4 (i.e., avoiding bias due to selective drop-out of level-5 and level-6 trials from less-balanced players).

Results are reported in three sections: (*1)* success rate, (*2)* log of *ap*-CoP and log of *ml*-CoP as well as their difference, that is log(*ap*/*ml*), (*3)* LSI computed for ap and ml (see formula 1). An increase in success rate, a negative change score (posttest minus pretest) of log(*ap*), log(*ml*), and log(*ap*/*ml*), or an increase in *ap-*LSI or *ml*-LSI reflect improvements in balance performance. In each section, we focus on the two interactions between pretest/posttest and group (contrast 1: training groups *vs.* control group; contrast 2: single-mode BT group *vs.* combined BT + PT group) and whether these interactions were moderated by balance conditions (static *vs.* dynamic), level of task difficulty, and—in the first two sections—non-dominant *vs.* dominant leg.

### Success rate

**Fig 3:**
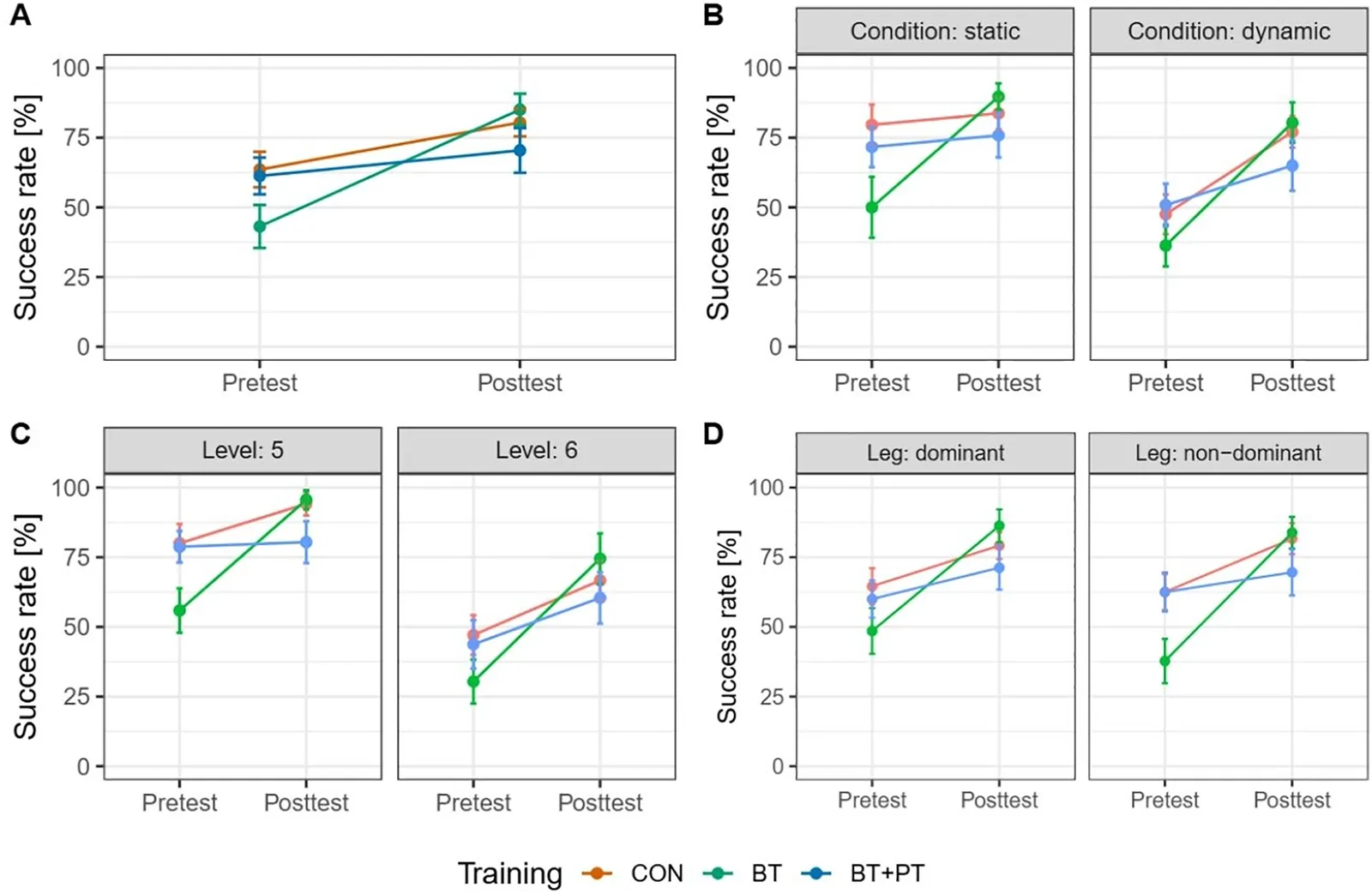
Success rate for the (A) group × time, (B) group × time × condition, (C) group × time × level, and (D) group × time × leg interaction. *Note:* Values are group means ± between-player standard errors with higher values indicating better balance performance. BT = unilateral single-mode balance training group; BT + PT = unilateral combined balance and plyometric jump training group; CON = active control group.

Table 1 presents all fixed main and interaction effects for the parameter success rate using the GLMM. Fig 3A and Fig 3B–D visualize the success rates for the two-factor (group × time) and three-factor (group × time × condition; group × time × level; group × time × leg) interactions, respectively. In general, success rate was significantly higher (*a)* at posttest *vs.* pretest (*b* = 2.540, *z* = 7.000, *p* < .001); (*b)* for the static *vs.* dynamic balance condition (*b* = 3.136, *z* = 5.159, *p* < .001); and (*c)* for task difficulty level 5 *vs.* 6 (*b* = −2.623, *z* = −11.884, *p* < .001). The main effect of leg was not significant. Aside from the above training-related interactions, there was a significant time × condition interaction (*b* = −.621, *z* = −2.426, *p* = .015), indicating that success rate improved more in the dynamic (Δ = .29) than in the static (Δ = .15) balance condition. In addition, there was a significant condition × level interaction (*b* = .695, *z* = 2.763, *p* = .006), indicating that the condition effect was larger for task difficulty level 6 (Δ = .23) compared to level 5 (Δ = .09).

**Table 1:** Fixed main and interaction effects of success rate for the generalized linear mixed model.

Term	Coef	SE	z	p
GM	1.597	0.570	2.801	.005
Time	**2.540**	**0.363**	**7.000**	**<.001**
Condition	**3.136**	**0.608**	**5.159**	**<.001**
Level	**−2.623**	**0.221**	**−11.884**	**<.001**
Leg	−0.197	0.141	−1.395	.163
Time × Condition	**−0.621**	**0.256**	**−2.426**	**.015**
Time × Level	−0.251	0.159	−1.578	.115
Time × Leg	0.118	0.091	1.292	.196
Condition × Level	**0.695**	**0.251**	**2.763**	**.006**
Condition × Leg	0.317	0.174	1.817	.069
Level × Leg	−0.075	0.094	−0.805	.421
TRNG *vs.* CON (= C1)	0.071	0.395	0.179	.858
C1 × Time	−0.015	0.247	−0.060	.952
C1 × Condition	0.092	0.423	0.218	.827
C1 × Level	−0.155	0.127	−1.218	.223
C1 × Leg	0.080	0.087	0.923	.356
C1 × Time × Condition	**−0.924**	**0.176**	**−5.266**	**<.001**
C1 × Time × Level	−0.003	0.113	−0.029	.977
C1 × Time × Leg	0.062	0.060	1.030	.303
BT *vs.* BT + PT (= C2)	−0.238	0.711	−0.335	.737
C2 × Time	**−1.050**	**0.452**	**−2.326**	**.020**
C2 × Condition	−0.148	0.754	−0.197	.844
C2 × Level	0.151	0.221	0.686	.493
C2 × Leg	0.298	0.159	1.876	.061
C2 × Time × Condition	**−0.816**	**0.303**	**−2.691**	**.007**
C2 × Time × Level	**0.384**	**0.194**	**1.982**	**.047**
C2 × Time × Leg	−0.170	0.114	−1.496	.135

*Note:* Coefficients are odds ratios estimated in the generalized linear mixed model. Bold values indicate statistically significant effects. BT = unilateral single-mode balance training group; BT + PT = unilateral combined balance and plyometric jump training group; CON = active control group; C1 = contrast 1; C2 = contrast 2; GM = grand mean; SE = standard error; TRNG = both training groups.

Further, intervention-related contrasts between the three groups revealed a significant C1 × time × condition interaction (*b* = −.924, *z* = −5.266, *p* < .001; Fig 3B) indicating that both training groups (i.e., single-mode BT group, combined BT + PT group) improved more compared to the active CON group in the dynamic balance condition and, in particular, in the static balance condition. The source of this interaction was primarily the significantly greater gains of the single-mode BT group rather than the combined BT + PT group in the static balance condition (C2: *b* = −.816, *z* = −2.691, *p* = .007; Fig 3B). In addition, the single-mode BT group improved significantly more than the combined BT + PT group in difficulty level 5 compared to level 6 (C2: *b* = .384, *z* = 1.982, *p* = .047; Fig 3C).

### Anteroposterior and mediolateral center of pressure indices

**Fig 4:**
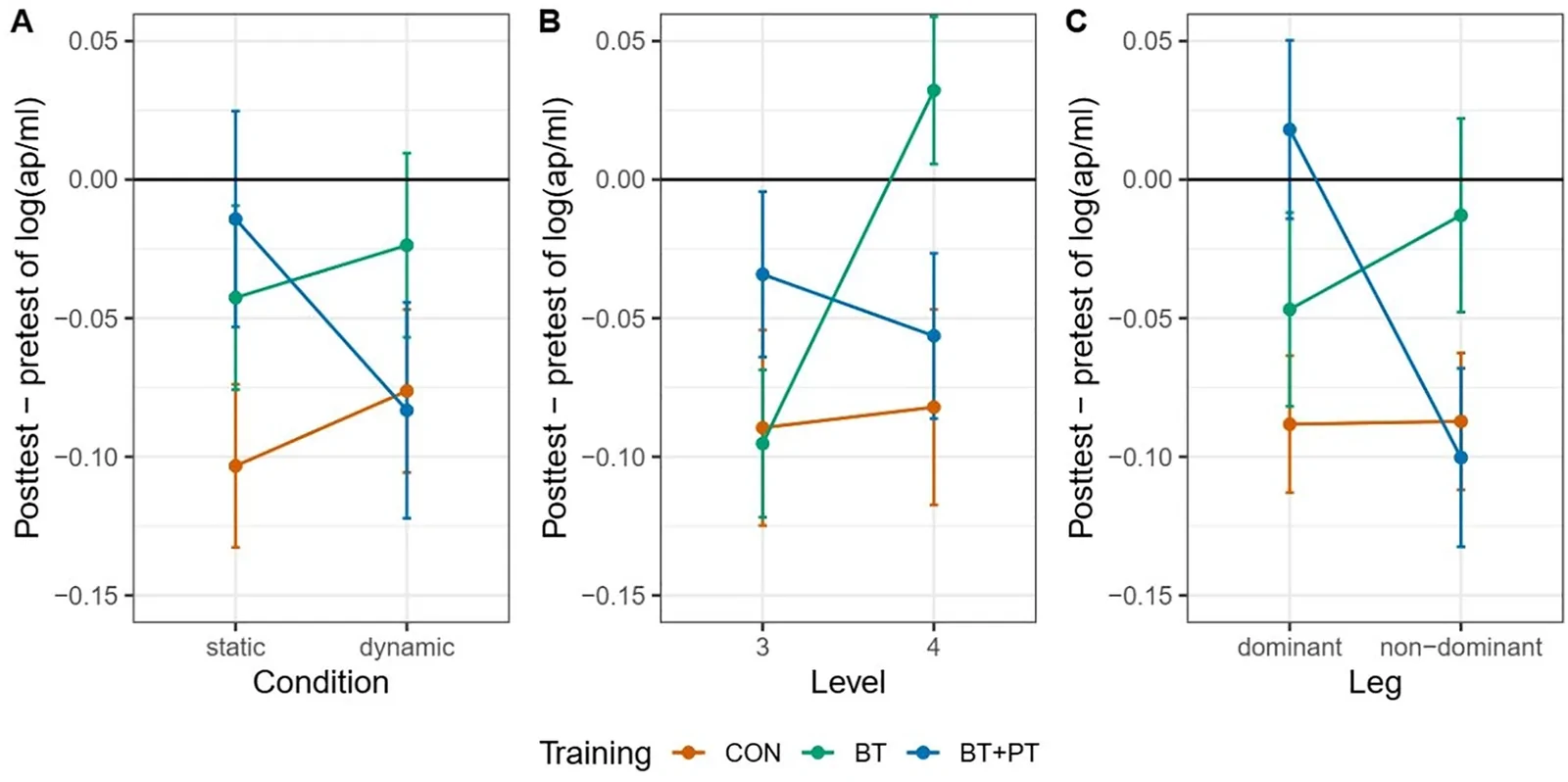
Anteroposterior-mediolateral center of pressure ratio for the (A) group × time × condition, (B) group × time × level, and (C) group × time × leg interaction plotted as change from pretest to posttest. *Note:* The zero line indicates no training-related change; the more negative the ratio, the larger the improvement from pretest to posttest. Values are group means ± within-players standard errors. ap = anteroposterior; BT = unilateral single-mode balance training group; BT + PT = unilateral combined balance and plyometric jump training group; CON = active control group; ml = mediolateral.

Table 2 presents the fixed main and interaction effects of CoP-based parameters using LMMs; Fig 4A–C visualize log(*ap*/*ml*) for the training-related three-factor interactions. In general, and as shown in the left columns of Table 2, there were *(a)* significant improvements from the pretest to the posttest (*b* = −.030, *z* = −3.268, *p* = .001), *(b)* significantly better performances for the static *vs.* dynamic balance condition (*b* = −.086, *z* = −6.373, *p* < .001), and *(c)* significantly better performances at task difficulty level 3 *vs.* 4 (*b* = .017, *z* = 2.187, *p* = .029). The main effect of leg was not significant.

**Table 2:** Fixed main and interaction effects of center of pressure-based indices for the linear mixed models.

Dependent variable:	log(*ap*/*ml*)			log(*ap*)			log(*ml*)		
Term	Coef	z	p	Coef	z	p	Coef	z	p
GM	0.443	32.860	<.001	2.724	121.797	.000	2.280	101.426	<.001
Time	**−0.030**	**−3.268**	**.001**	**−0.030**	**−2.742**	**.006**	0.001	0.076	.939
Condition	**−0.086**	**−6.373**	**<.001**	**−0.203**	**−9.201**	**.000**	**−0.117**	**−5.992**	**<.001**
Level	**0.017**	**2.187**	**.029**	**0.032**	**4.307**	**.000**	**0.015**	**2.576**	**.010**
Leg	−0.012	−1.475	.140	0.001	0.168	.866	0.013	1.933	.053
Time × Condition	0.007	0.635	.525	−0.014	−1.504	.133	**−0.021**	**−2.582**	**.010**
Time × Level	0.008	1.502	.133	**0.013**	**2.672**	**.008**	0.005	1.153	.249
Time × Leg	−0.008	−1.548	.122	−0.007	−1.408	.159	0.001	0.288	.774
Condition × Level	−0.013	−1.225	.221	−0.009	−0.919	.358	0.004	0.473	.636
Condition × Leg	−0.003	−0.276	.783	−0.007	−0.735	.462	−0.005	−0.555	.579
Level × Leg	−0.002	−0.345	.730	−0.003	−0.573	.566	−0.001	−0.226	.822
TRNG *vs.* CON (= C1)	0.003	0.340	.734	**0.032**	**2.051**	**.040**	0.029	1.850	.064
C1 × Time	−0.005	−0.759	.448	−0.002	−0.227	.821	0.003	0.435	.663
C1 × Condition	−0.002	−0.189	.850	0.004	0.232	.816	0.005	0.379	.705
C1 × Level	−0.005	−1.368	.171	0.001	0.145	.885	**0.006**	**2.033**	**.042**
C1 × Leg	−0.002	−0.546	.585	−0.003	−0.654	.513	−0.000	−0.051	.959
C1 × Time × Condition	−0.008	−1.124	.261	−0.005	−0.796	.426	0.003	0.528	.598
C1 × Time × Level	−0.004	−0.968	.333	−0.002	−0.560	.576	0.002	0.580	.562
C1 × Time × Leg	0.004	1.044	.296	0.005	1.525	.127	0.001	0.439	.660
BT *vs.* BT + PT (= C2)	0.004	0.236	.814	−0.018	−0.639	.523	−0.021	−0.746	.456
C2 × Time	−0.017	−1.516	.130	−0.024	−1.800	.072	−0.008	−0.639	.523
C2 × Condition	0.002	0.137	.891	−0.029	−1.071	.284	−0.032	−1.334	.182
C2 × Level	0.010	1.373	.170	0.005	0.749	.454	−0.004	−0.806	.420
C2 × Leg	−0.005	−0.719	.472	−0.005	−0.741	.459	0.000	0.024	.980
C2 × Time × Condition	**0.026**	**1.991**	**.046**	**0.033**	**2.781**	**.005**	0.008	0.751	.453
C2 × Time × Level	**−0.018**	**−2.723**	**.006**	**−0.025**	**−4.283**	**<.001**	−0.008	−1.499	.134
C2 × Time × Leg	**−0.017**	**−2.574**	**.010**	−0.006	−1.092	.275	**0.010**	**2.034**	**.042**

*Note:* Coefficients are fixed effect estimates in the linear mixed models. Bold values indicate statistically significant effects. ap = anteroposterior; BT = unilateral single-mode balance training group; BT + PT = unilateral combined balance and plyometric jump training group; CON = active control group; C1 = contrast 1; C2 = contrast 2; GM = grand mean; ml = mediolateral; TRNG = both training groups.

Further, there were significant C2 × time × condition (*b* = .026, *z* = 1.991, *p* = .046; Fig 4A), C2 × time × level (*b* = −.018, *z* = −2.723, *p* = .006; Fig 4B), and C2 × time × leg (*b* = −.017, *z* = −2.574, *p* = .010; Fig 4C) interactions indicating that the combined BT + PT group improved more (*a)* in the dynamic *vs.* static balance condition, (*b)* in task difficulty level 4 *vs.* 3, and (*c)* in the non-dominant *vs.* dominant leg whereas the trend was always in the opposite direction for the single-mode BT group.

We also estimated the LMM separately for log(*ap*) and log(*ml*) to determine the primary source of the significant three-factor interactions. As shown in the middle and right columns of **Table 2**, there were significant C2 × time × condition (*ap*: *b* = .033, *z* = 2.781, *p* = .005), C2 × time × level (*ap*: *b* = −.025, *z* = −4.283, *p* < .001), and C2 × time × leg (*ml*: *b* = .010, *z* = −2.034, *p* = .042) interactions. This indicates that the aforementioned improvements with respect to condition and difficulty level are primarily determined by the ap-direction, while those regarding leg are attributable to the ml-direction. Corresponding interactions for C1 (i.e., training groups *vs.* control group) with time and the factors condition, level, and leg were not significant.

### Anteroposterior and mediolateral limb symmetry index indices

As a follow-up to the significant training-related (log)*ap-* and (log)*ml-*CoP interactions reported before, we estimated LSI effects for the two CoP-based indices. There was a total of 447 of 456 measures (i.e., 57 players × 2 times [pretest, posttest] × 2 conditions [static, dynamic] × 2 levels [3 and 4]). Nine measures were missing because of three invalid trials for one leg in one of the eight conditions. They came from three players with one and three players with two missing values. LMMs varying only GMs (i.e., intercepts) fit best for CoP-based LSI values.

**Fig 5:**
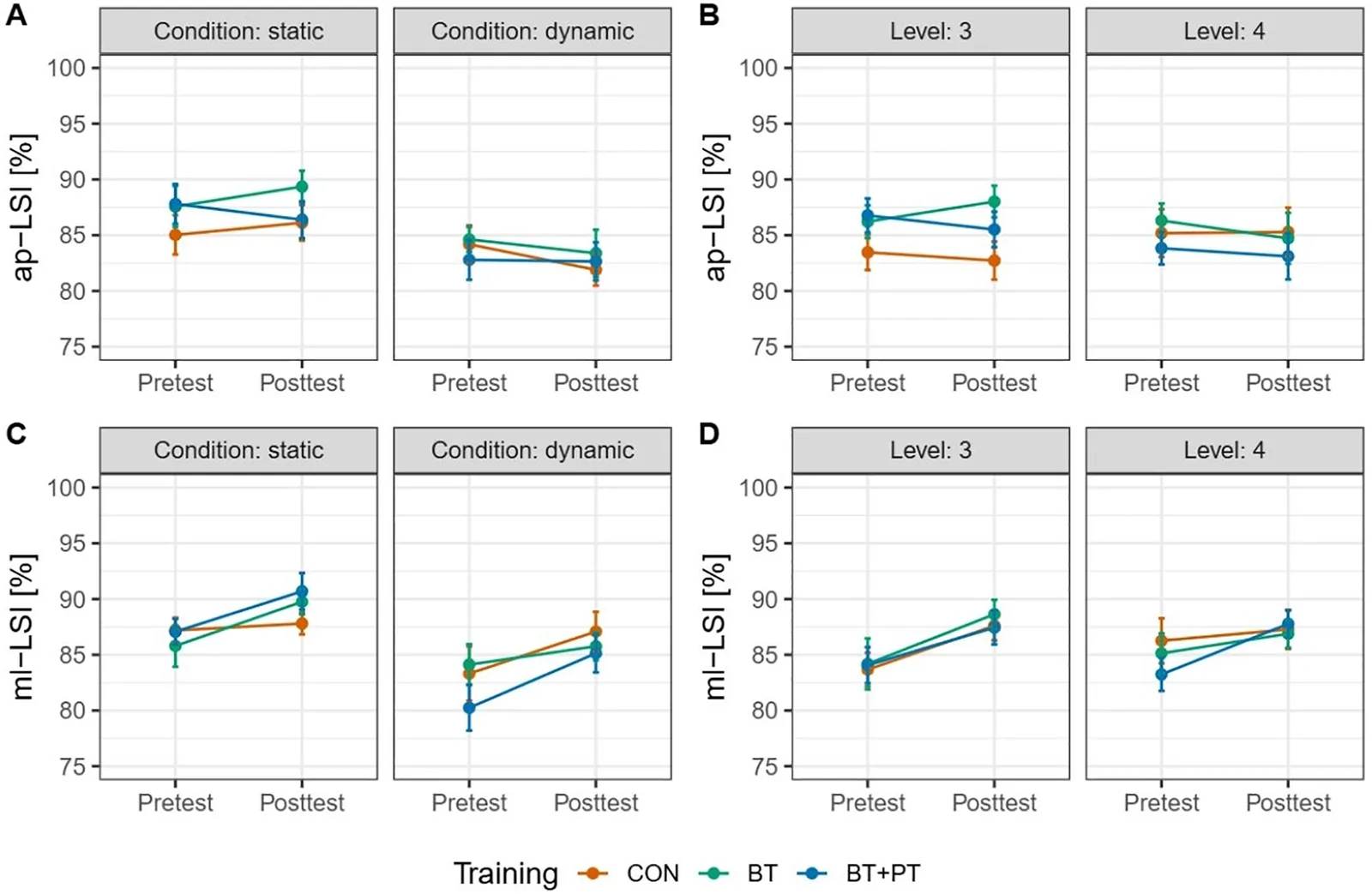
Anteroposterior limb symmetry index for (A) group × time × condition, (B) group × time × level; corresponding interactions for the mediolateral limb symmetry index are shown in (C) and (D). *Note:* Values are group means ± between-players standard errors with higher values indicating larger interlimb symmetry (≈ lower interlimb asymmetry). ap = anteroposterior; BT = unilateral single-mode balance training group; BT + PT = unilateral combined balance and plyometric jump training group; CON = active control group; ml = mediolateral; LSI = limb symmetry index.

Table 3 presents the fixed main and interaction LSI effects for the two LMMs; Fig 5A–B and Fig 5C–D visualize the two training-related three-factor interactions for *ap*-LSI and *ml*-LSI, respectively. None of the interactions were significant. However, there was a significant overall *ml*-LSI improvement from pretest to posttest (*b* = −5.410, *z* = −2.056, *p* = .040). Although not significant, there was also a numerical trend towards higher LSI in the static than the dynamic condition for *ap*-LSI (*b* = 4.220, *z* = 1.818, *p* = .069)*.*

**Table 3:** Fixed main and interaction effects of center of pressure-based limb symmetry index indices for the linear mixed models.

		*ap*-LSI			*ml*-LSI	
Term	Coef	z	p	Coef	z	p
GM	80.616	39.856	.000	87.243	46.980	.000
Time	2.424	0.851	.395	**−5.410**	**−2.056**	**.040**
Condition	**4.220**	**1.818**	**.069**	0.719	0.335	.737
Level	2.564	1.105	.269	−0.323	−0.151	.880
Time × Condition	−3.371	−1.020	.308	2.963	0.971	.332
Time × Level	−0.232	−0.070	.944	3.626	1.188	.235
TRNG *vs.* CON (= C1)	2.986	1.042	.298	−2.004	−0.762	.446
C1 × Time	−1.760	−0.437	.662	0.832	0.223	.823
C1 × Condition	−0.506	−0.153	.878	4.840	1.585	.113
C1 × Level	−4.462	−1.351	.177	0.108	0.035	.972
C1 × Time x Condition	4.670	0.999	.318	−1.700	−0.394	.694
C1 × Time x Level	−0.816	−0.175	.861	−4.243	−0.983	.326
BT *vs.* BT + PT (= C2)	4.417	1.480	.139	−0.610	−0.223	.824
C2 × Time	−2.929	−0.691	.489	2.159	0.552	.581
C2 × Condition	1.751	0.511	.609	3.275	1.035	.301
C2 × Level	−5.862	−1.712	.087	−1.424	−0.450	.653
C2 × Time x Condition	0.219	0.045	.964	−5.127	−1.131	.258
C2 × Time x Level	3.966	0.808	.419	−0.723	−0.159	.873

*Note:* Coefficients are fixed effect estimates in the linear mixed models. Bold values indicate statistically significant effects. ap = anteroposterior; BT = unilateral single-mode balance training group; BT + PT = unilateral combined balance and plyometric jump training group; CON = active control group, C1 = contrast 1; C2 = contrast 2; GM = grand mean; ml = mediolateral; LSI = limb symmetry index; TRNG = both training groups.

## Discussion

The aim of this study was to investigate the effects of single-mode BT or combined BT + PT compared with an active CON group on young male soccer players’ interlimb static and dynamic balance performance. Thus, the present study represents an applied extension of a previous investigation [24] on the effects of single-mode BT versus combined BT + PT on proactive and reactive balance performance as well as muscle activity in young male soccer players. Our first assumption—that both training modalities lead to improved interlimb static and dynamic postural control—was confirmed by the significant time × condition interaction for the parameter success rate. In the *first step of the statistical analysis*, this binary parameter indicates whether the static or dynamic balance task was successfully completed at each difficulty level or not, regardless of quality of postural control (i.e., small or large CoP displacements). Accordingly, the number of soccer players who successfully completed the static and dynamic balance task increased more strongly in both training groups compared with the active CON group. This finding is consistent with other studies [49,50] that reported performance improvements in soccer players’ postural control following BT. For instance, Heleno et al. [49] implemented a five-week BT program in addition to regular soccer training in young male players aged 14–16 years. Compared with an active CON group that performed regular soccer training only, the BT group showed significant improvements in balance performance (i.e., increased reach distances in the Star Excursion Balance Test). Further, Gioftsidou and colleagues [50] applied BT before or after regular soccer training and observed significantly decreased CoP displacements in both training groups compared to the active CON group (i.e., soccer training only).

Concerning our second assumption—that combined BT + PT would elicit greater improvements than single-mode BT — the results differed depending on the parameter considered. Contrary findings were observed for the parameter success rate, whereas the results for the CoP-based indices were consistent with our hypothesis. Precisely, participants of the single-mode BT group showed significantly greater gains in success rate compared with those in the combined BT + PT group. This finding was particularly observed for the static balance condition and can be explained by the principle of training specificity [51,52]. Accordingly, improvements in postural control following single-mode BT are specific to the training conditions. In this regard, players in the single-mode BT group completed a greater volume of balance exercises than those in the combined BT + PT group, as they completed four instead of two sets per exercise. Moreover, they performed proportionally fewer dynamic movements due to the absence of plyometric exercises. Both factors may have contributed to the greater improvements observed in the single-mode BT group with respect to the static balance condition. In addition, the single-mode BT group improved significantly more than the combined BT + PT group at difficulty level 5 compared to level 6. Accordingly, the requirements for mastering difficulty level 5 were appropriate to enable further improvements in balance performance. In contrast, the demands of difficulty level 6 may have been too challenging for the players in the combined BT + PT group due to their lower volume of balance exercises mentioned above, possibly leading to a “floor effect” that masked training-induced adaptations.

Consistent with our second assumption, the combined BT + PT group showed significantly greater gains in the CoP-based indices compared to the single-mode BT group. This finding is in line with previous work [16,18,19] that also reported improvements in postural control as a result of combining balance and plyometric exercises. For instance, Al Attar and Husain [18] allocated athletes to a combined BT + PT group, a single-mode BT group, a single-mode PT group or a CON group. Following six weeks of training, all training groups demonstrated improvements in balance performance (i.e., increased limits of stability) compared with the CON group. Moreover, the combined BT + PT group exhibited greater gains than both single-mode training groups. Similarly, Bouteraa et al. [16] examined the effects of eight weeks of combined BT + PT on physical fitness outcomes in female basketball players aged ≈ 16 years and found improvements in balance performance (i.e., increased reach distances in the Y Balance Test and stance duration on the unipedal stance test) compared to an active CON group (i.e., basketball training only). The observation that players in the combined BT + PT group showed greater improvements compared with those in the single-mode BT group could be attributed to specific but complementary adaptive mechanisms [20,21]. Precisely, balance exercises primarily influence the afferent input to the motoneuron, whereas plyometric exercises affect intramuscular coordination on the efferent pathway [53,54]. Both mechanisms enhance postural control and were addressed in the combined BT + PT group, while only the first mechanism was addressed in the single-mode BT group.

In addition, greater improvements (i.e., a larger negative *ap/ml* ratio) were particularly detected in the dynamic balance condition, which in turn can be explained by the principle of training specificity [51,52]. In the combined BT + PT group, the inclusion of plyometric jump exercises during training resulted in a greater proportion of compensatory movements required to maintain and restore balance, which may have promoted the larger adaptations observed in the dynamic balance condition. This interpretation is further supported by the greater improvements observed at the higher difficulty level 4 versus level 3, as this level imposes greater demands in terms of compensatory movements for successful task completion.

Why were larger effects detected for the combined BT + PT group in terms of the CoP-based indices and for single-mode BT with regard to the success rate? The CoP-based indices resulted from the *second step of statistical analysis* and, in contrast to the binary and therefore relatively “rough” parameter success rate, allow a more in-depth analysis of adaptive processes. In this regard, there is evidence—as mentioned before—that combined BT + PT induces complementary adaptations, whereas single-mode BT induces only limited adaptations [20,21,53,54]. Based on this, differences in the sensitivity of the parameters used could be the reason for the diverging intervention effects. In other words, the success rate has lower sensitivity and allows the detection of singular adaptations, whereas the CoP-based indices have higher sensitivity and enable the detection of combined adaptations.

The CoP-based parameters not only allow for a more in-depth analysis of adaptation processes but also enable conclusions about direction-specific effects. The two findings mentioned above (i.e., greater improvements in the dynamic balance condition and at the higher difficulty level) were mainly driven by changes in the ap-direction, in which continuous leg-swing movements were performed during dynamic balance assessment. Accordingly, direction-specific adaptations appear to support stability. In addition, significantly greater gains were observed for the combined BT + PT group compared to the single-mode BT group, particularly in the non-dominant leg (i.e., the leg used to perform unilateral training exercises). This again implies adaptations in accordance with the principle of training specificity [51,52]. However, these adaptations were mainly determined by the ml-direction. A possible explanation emerges from research [55] reporting greater postural sway in the ml- compared to the ap-direction when standing on a narrow surface. This indicates that the ml-direction is mechanically less stable and therefore allows more room (greater adaptive reserve) for training-related improvements in postural control.

In the *third step of statistical analysis*, the two legs were no longer considered separately but were directly related to each other by calculating LSI indices. Regardless of the training modality, a statistically significant increase in LSI values from ≈85% (pretest) to ≈90% (posttest) was observed (see Fig 5C), indicating a clinically meaningful shift from interlimb asymmetry (>10%) to interlimb asymmetry (≤10%) in postural control. This finding is in accordance with previous literature [9–11]. For instance, Rasool and George [9] conducted a four-week BT program in adult athletes and detected significantly increased reach distances in the Y Balance Test in the trained limb and, to a lesser extent, in the untrained limb for the training but not for the CON group. Further, Oliveira and colleagues [10] showed significant enhancements in postural sway in the trained leg, and smaller improvements in the untrained leg, for the training compared to the CON group. Although no LSI indices were calculated in either study, the reported findings suggest an increase in interlimb symmetry.

This study has some limitations that should be considered when interpreting the findings. First, this study does not allow conclusions to be drawn about adult soccer players, as it focused exclusively on young players. Second, the findings cannot be generalized to female soccer players since only male players were included. Third, the sample consisted solely of highly trained soccer players (Tier level 3), which restricts the applicability of the results to other playing levels. Fourth, the intervention program completed by the active control group might not have been entirely neutral since stretching exercises can also have beneficial effects on proprioception and balance performance [30]. Fifth, no internal training load parameters were measured. Therefore, future studies should be expanded to include the measurement of metabolic (e.g., heart rate, blood lactate) and biomechanical (e.g., joint contact forces, muscle-tendon forces) components. Sixth, between-trial test-retest reliability was poor to moderate for the dynamic balance task. Therefore, it cannot be ruled out that the low repeatability of data collection may have affected the accuracy of the present findings. Seventh, training-related changes were determined on a performance level but not in terms of injury risk. Consequently, future studies are needed that investigate the relationship between training-related reductions in interlimb asymmetry and injury risk in young soccer players. Eighth, although the total training volume was distributed equally among the groups, the proportion of the additional intervention (i.e., 60 minutes per week) was smaller than the proportion of soccer-specific training (360–420 minutes per week) and athletic training (90–180 minutes per week). Therefore, it cannot be assumed that the observed effects were caused solely by the intervention. Ninth, the obtained findings resulted from complex interactions among several independent variables (e.g., group × time × condition/level/leg) based on partially overlapping data (i.e., log [*ap/ml*] and LSI), which compromises their meaning and interpretation from a physiological perspective. Furthermore, some findings are close to the conventional *p* = 0.05 threshold, which increases the likelihood of spurious and non-replicable results.

## Conclusions

The present study adds further applied evidence to previous investigations of training-related changes on interlimb asymmetry. Depending on the balance outcome considered, we found that the binary parameter success rate (obtained from a superficial analysis) was improved following single-mode BT and the metric parameter log (*ap/ml*) CoP following combined BT + PT (obtained from more in-depth analyses). We additionally detected that both training modalities yielded in enhanced interlimb symmetry. These observations expand upon existing research in athletes, providing important new insight into the effects of both training modalities on interlimb static and dynamic postural control in young male soccer players. The floor and ceiling effects across outcome domains prevent definitive conclusion regarding the superiority of either training modality. However, the detailed analyses based on CoP-derived indices favor the combined BT + PT approach, suggesting that this training modality is particularly effective in enhancing balance regulatory processes.

### Generative AI statement

Gemini Pro 3 and Claude Sonnet 4.5 were used for assistance with coding of Julia scripts for production of tables and R scripts for figures. Further, ChatGPT (OpenAI, San Francisco, CA) was used to create Figure 2.

## Supporting information

S1 Fig Box-Cox lambda for anteroposterior and mediolateral root mean square. (DOCX)

S2 Fig q-q plots for log(*ap/ml*), log(*ap*), and log(*ml*). (DOCX)

S1 Table Random-effect structures for generalized linear mixed model and three linear mixed models. (DOCX)
