# Supplementary material for: Effects of unilateral single-mode balance training compared to combined balance and plyometric training on soccer players’ interlimb asymmetry in static and dynamic balance performance
Source: PLoS One. 2026 Sep 16;21(9):e0358565. doi: 10.1371/journal.pone.0358565 (PMC13581024; doi:10.1371/journal.pone.0358565)
Supplement: Supplementary file 1 [file pone.0358565.s001.docx]

**Supporting information**

**Figure S1.** Box-Cox lambda for anteroposterior and mediolateral root mean square.
