# Supplementary material for: Effects of unilateral single-mode balance training compared to combined balance and plyometric training on soccer players’ interlimb asymmetry in static and dynamic balance performance
Source: PLoS One. 2026 Sep 16;21(9):e0358565. doi: 10.1371/journal.pone.0358565 (PMC13581024; doi:10.1371/journal.pone.0358565)
Supplement: Supplementary file 3 [file pone.0358565.s003.docx]

**Supporting information**

**Table S1.** Random-effect structures for generalized linear mixed model and three linear mixed models.

| **Parameter** | **Type** | **Success** | **log(*ap/ml*)** | **log(ap)** | **log(*ml*)** |
| --- | --- | --- | --- | --- | --- |
| GM | SD | 4.052 | 0.079 | 0.155 | 0.163 |
| Time | SD | 2.267 | 0.040 | 0.064 | 0.058 |
| Condition | SD | 3.852 | 0.055 | 0.144 | 0.133 |
| Level | SD | 0.517 |  |  | 0.010 |
| Leg | SD | 0.641 |  |  | 0.028 |
| GM & Time | Corr | 0.386 |  |  | -0.103 |
| GM & Condition | Corr | -0.173 |  |  | -0.314 |
| GM & Level | Corr | 0.869 |  |  | 0.773 |
| GM & Leg | Corr | 0.401 |  |  | 0.518 |
| Time & Condition | Corr | -0.211 |  |  | 0.102 |
| Time & Level | Corr | 0.209 |  |  | 0.513 |
| Time & Leg | Corr | 0.012 |  |  | -0.363 |
| Condition & Level | Corr | 0.253 |  |  | 0.005 |
| Condition & Leg | Corr | 0.429 |  |  | -0.061 |
| Level & Leg | Corr | 0.396 |  |  | 0.243 |
| Residual | SD | NA | 0.272 | 0.247 | 0.213 |

*Note.* Corr = correlation parameter; GM = grand mean; NA = not available, i.e. there is no residual estimate for the GLMM; SD = square root of variance.
