# Supplementary material for: Effects of unilateral single-mode balance training compared to combined balance and plyometric training on soccer players’ interlimb asymmetry in static and dynamic balance performance
Source: PLoS One. 2026 Sep 16;21(9):e0358565. doi: 10.1371/journal.pone.0358565 (PMC13581024; doi:10.1371/journal.pone.0358565)
Supplement: Supplementary file 4 [file pone.0358565.s005.docx]

Manuscript Number: **PONE-D-26-01855**

Title: **Interlimb static and dynamic postural control in young soccer players: Role of training**

**Responses to Journal Requirements:**

**Comment 1: Please ensure that your manuscript meets PLOS ONE's style requirements, including those for file naming.**

Response: We have checked the PLOS ONE's style requirements and confirm that they are met including those for file naming.

**Comment 2: 2. Thank you for stating the following financial disclosure:**

**“This study was funded by a grant from the Deutsche Forschungsgemeinschaft (DFG; German Research Foundation) – grant numbers: MU 3327/5-1 and PA 774/21-1. The support by the Open Access Publication Fund of the University of Duisburg-Essen is acknowledged. The funding body is independent of the design of the study and collection, analysis, and interpretation of data and in writing the manuscript. Open access funding enabled and organized by the project DEAL.” Please state what role the funders took in the study.  If the funders had no role, please state: "The funders had no role in study design, data collection and analysis, decision to publish, or preparation of the manuscript." If this statement is not correct you must amend it as needed. Please include this amended Role of Funder statement in your cover letter; we will change the online submission form on your behalf.**

Response: We hereby confirm that the funders had no role and have confirmed this in the cover letter.

**Comment 3: Please note that funding information should not appear in any section or other areas of your manuscript. We will only publish funding information present in the Funding Statement section of the online submission form. Please remove any funding-related text from the manuscript.**

Response: In accordance with your suggestion, we have removed any funding-related text from the manuscript.

**Comment 4: Your ethics statement should only appear in the Methods section of your manuscript. If your ethics statement is written in any section besides the Methods, please delete it from any other section.**

Response: In accordance with your suggestion, we now only provide the ethics statement in the Material and Method section (cf. Participants) as follows: “*Ethical permission (approval number: TM_04.06.2020) was given by the Human Ethics Committee at the University of Duisburg-Essen, Faculty of Educational Sciences. Participants’ assent and parents’ written informed consent were obtained prior to the start of the study.”* This change and all other changes in response to the comments related to Journal Requirements are highlighted in purple.

**Comment 5: Please upload a new copy of Figures 1, 3, 4, and 5 as the detail is not clear.**

Response: In accordance with your suggestion, we have uploaded a new copy of Figures 1, 3, 4, and 5.

**Comment 6: We note that Figure 2 includes an image of a participant in the study.**

Response: The participant shown in Figure 2 does not represent an image of a participant in the study but was created by using ChatGPT (OpenAI, San Francisco, CA). This information has been added to the Material and Method section (cf. Participants) as follows: *“The individual shown in this diagram was created by using ChatGPT (OpenAI, San Francisco, CA).”* and is now mentioned in the Generative AI statement*: “Further, ChatGPT (OpenAI, San Francisco, CA) was used to create Figure 2.”*

**Comment 7: We note that Figure 2 in your submission contains copyrighted images. All PLOS content is published under the Creative Commons Attribution License (CC BY 4.0), which means that the manuscript, images, and Supporting Information files will be freely available online, and any third party is permitted to access, download, copy, distribute, and use these materials in any way, even commercially, with proper attribution.**

Response: As stated in response to the previous comment, Figure 2 was created by using ChatGPT (OpenAI, San Francisco, CA) and does not contain copyrighted images.

**Comment 8: If the reviewer comments include a recommendation to cite specific previously published works, please review and evaluate these publications to determine whether they are relevant and should be cited. There is no requirement to cite these works unless the editor has indicated otherwise.**

Response: The reviewers’ comments do not include a recommendation to cite specific previously published works.

**Responses to Reviewer #1:**

**Comment: This manuscript investigates the effects of unilateral training modalities on interlimb asymmetry in static and dynamic postural control in elite youth soccer players. The study is conducted within a high-level academy setting, the intervention is well structured, and implementation during the competitive season represents a clear applied strength. The topic is relevant, particularly in the context of neuromuscular performance and asymmetry modulation in youth sport. The dataset appears carefully collected and the study is generally well organized.**

**However, several conceptual, methodological, and transparency-related issues require clarification and strengthening before the manuscript can be considered for publication.**

**For these reasons, I recommend major revision.**

**The required revisions primarily concern:**

**• transparency regarding overlap with previously published data,**

**• conceptual justification of asymmetry reduction,**

**• statistical handling of cluster randomization and maturation,**

**• cautious interpretation of practical relevance,**

**• and a more conservative framing of the discussion and conclusions.**

Response: We thank the reviewer for summarizing the manuscript and for this affirmative comment. Further, we are grateful for the time and effort invested in providing constructive criticism. Below you will find the responses to your comments. All changes made in response to your comments were highlighted in yellow in the revised manuscript.

**Major Comments**

**Transparency regarding previously published data.**

**A closely related study by the same authors using the identical intervention cohort (elite youth soccer players from SG Dynamo Dresden; 9-week unilateral BT vs. BT+PT vs. control; YBT-LQ, SLDL, EMG; LSI-based analyses) has recently been published.**

**Given the apparent overlap in:**

**• population,**

**• intervention design,**

**• measurement tools,**

**• and asymmetry-based outcome analyses,**

**it is essential that the authors explicitly clarify:**

**1. Whether the present manuscript uses the same dataset.**

**2. If so, which analyses are novel and how they substantively differ from the previously published work.**

**3. That the prior publication is transparently cited and clearly distinguished.**

**This is not inherently problematic provided that the research questions and analytical focus are clearly distinct. However, explicit clarification is required to avoid concerns regarding redundant publication or insufficient differentiation between manuscripts.**

Response: We apologize for being imprecise. The dataset in this manuscript with *N*=57 participants is similar but not identical to that from a previous study with *N*=64 participants [1], as it includes only some of the same participants. This is because not all participants from the previous study were able to complete the progressively increasing task difficulty in the present study. In other words, the population is similar, the intervention design is identical, but the measurement tools (present study: unipedal stance with/without leg swing vs. previous study: Y Balance Test – Lower Quarter, Single leg drop landing test) and the asymmetry-based outcome analyses (present study: success rate, posttest – pretest of log, ap-/ml-LSI vs. previous study: YBT-LQ-/SLDL-LSI) are different. For clarification, we have added the following information to the Material and Methods section (cf. Data analyses): *“Some of the data originate from a previously published study [1] in which the same interventions were used but different assessments were conducted.”*

**Comment: Conceptual justification of interlimb asymmetry reduction.**

**The manuscript implicitly assumes that reducing interlimb asymmetry is beneficial. This assumption requires stronger theoretical grounding.**

**Key issues that should be addressed:**

**• Are asymmetries inherently maladaptive in elite youth soccer?**

**• Could certain asymmetries be functionally adaptive due to sport-specific demands (e.g., dominant kicking leg vs. stance leg)?**

**• Is there direct evidence that reducing asymmetry improves performance or reduces injury risk specifically in elite youth soccer players?**

**• What is the baseline asymmetry magnitude in this cohort, and is it clinically meaningful?**

**Currently, the injury-risk argument relies largely on basketball or mixed-athlete populations. Transferability to elite youth soccer requires more careful discussion.**

**A more differentiated perspective on adaptive vs. maladaptive asymmetries would substantially strengthen the theoretical framework.**

Response: The studies by Grindem et al. [2] and Logerstedt et al. [3] indicate that interlimb asymmetry values >10% are clinically meaningful, and it seems reasonable to consider this threshold as an indication that deviates from the normal pattern and is therefore maladaptive.

Further, we agree with the reviewer that interlimb asymmetry in postural control arises, among others, from the long-standing preferential use of one limb to perform balance requirements [4], and due to the fact that passing and kicking in soccer are preferably performed with the same leg while keeping balance with the other leg, it is referred to as “functional asymmetry” [5]. However, Gonzalez-Fernandez et al. [6] reported that even amateur soccer players achieve high levels of interlimb asymmetry (i.e., 5.3–12.0%). Accordingly, interventions aiming to reduce interlimb asymmetry should be conducted at an early stage (in youth athletes).

There is evidence that interventions have the potential to reduce interlimb asymmetry [7] and improve neuromuscular parameters [8]. However, no such study exists for soccer. Therefore, as part of a project funded by the German Research Foundation (grant numbers: MU 3327/5-1 and PA 774/21-1), we have investigated the role of single-mode versus combined training on youth soccer players’ interlimb asymmetry in balance and neuromuscular performance. The results showed significant improvements in interlimb asymmetry and muscle activity [1]. The present manuscript continues this line of research and analyses, using a three-step approach (first step: binary parameters; second step: CoP-based indices; third step: LSI indices), the effects of single-mode versus combined training on youth soccer players’ interlimb asymmetry in static and dynamic balance performance. However, future studies are needed to confirm or reject our findings and to address additional aspects (e.g., fatigue). In the latter case, a proposal for a follow-up study has been submitted to the German Research Foundation and is currently under review.

To date, there is no evidence for a link between reduced interlimb asymmetry and injury risk in elite youth soccer players. This is likely because such findings would require epidemiological studies with relatively large sample sizes conducted over a long period of time. We have addressed this point in the Limitations section as follows: *“Fifth, training-related changes were determined on a performance level but not in terms of injury risk. Consequently, future studies are needed that investigate the relationship between training-related reductions in interlimb asymmetry and injury risk in young soccer players.”*

In terms of baseline asymmetry magnitude in this cohort, we have revised our previous statement in the Discussion section as follows *“Regardless of the training modality, a statistically significant increase in LSI values from ≈85% (pretest) to ≈90% (posttest) was observed (see Fig 5 C), indicating a clinically meaningful shift from interlimb asymmetry (>10%) to interlimb asymmetry (≤10%) in postural control.”*

With regard to the injury-risk argument, we have now reported findings from a study [9] that investigated different sports including soccer.

**Comment: Practical and clinical relevance**

**Although statistically significant reductions in LSI were observed, the practical relevance remains unclear.**

**Please clarify:**

**• Did the changes move players below established risk thresholds?**

**• Are the observed reductions large enough to meaningfully influence injury risk?**

**• Were injury data analyzed in relation to asymmetry changes?**

**At present, the conclusions may overstate practical implications. A clearer distinction between statistical significance and applied relevance is necessary.**

Response: Thank you for the valuable feedback. In terms of risk threshold, we have revised our statement in the Discussion section as follows *“Regardless of the training modality, a statistically significant increase in LSI values from ≈85% (pretest) to ≈90% (posttest) was observed (see Fig 5 C), indicating a clinically meaningful shift from interlimb asymmetry (>10%) to interlimb asymmetry (≤10%) in postural control.”*

Concerning your question, whether the observed reductions are large enough to be rated as meaningfully with respect to injury risk, we refer to the findings reported by Grindem et al. [2] and Logerstedt et al. [3] as their results suggest that interlimb asymmetries exceeding 10% are clinically meaningful. Accordingly, it appears justified to interpret this threshold as a deviation from typical movement patterns and thus may influence injury risk.

However, we did not analyze injury data in relation to changes in interlimb asymmetry, which is an important aim for future studies. Consequently, we have addressed this point in the Limitations section as follows: *“Fifth, training-related changes were determined on a performance level but not in terms of injury risk. Consequently, future studies are needed that investigate the relationship between training-related reductions in interlimb asymmetry and injury risk in young soccer players.”*

Further, we have revised the conclusions both in the Abstract and the Manuscript as follows.

Cf. Abstract: *“[…] Given the greater improvements in parameters obtained from the more in-depth analyses (i.e., CoP-based indices) in favor of the combined BT+PT, this training modality seems to be more suitable for enhancing interlimb postural control. From a practical perspective, the improvements in CoP-based indices suggest that processes of balance regulation in particular benefit from the combined BT+PT.”*

Cf. Manuscript: “*[…] The greater effectiveness of combined BT+PT in parameters resulting from more sophisticated, in-depth analyses suggest that this training modality is probably better suited to positively influencing interlimb postural control.”*

**Comment: Cluster randomization and statistical modelling**

**The manuscript states that cluster randomization was applied.**

**However, the statistical analyses appear to rely on repeated measures ANOVA without modelling potential cluster effects.**

**Please clarify:**

**• What defined a cluster (team, age group, subgroup)?**

**• Was clustering accounted for statistically?**

**• If not, provide justification or consider reanalysis using appropriate mixed-effects modelling.**

**Failure to account for clustering may affect inference validity.**

Response: We apologize for lack of precision. Players came from three age groups (squads) from one team (i.e., SG Dynamo Dresden e.V.). With “cluster randomization” we referred to the random assignment of groups of 6–8 players per age group (squad) to one of the three interventions. This approach was used to ensure an even distribution of players across age categories and thus to rule out the influence of chronological age. Thus, neither team nor age group nor randomization cluster could be included as a random factor in the (G)LMMs; they would have only 1, 3, and levels, respectively. For clarification, we added the following information to the Material and Methods section (cf. Participants): *“To guard against potential confounding effects of between-subject differences in chronological age, maturity offset, and intervention type, we randomly assigned groups of 6–8 players per age group (i.e., U13, U15, U17 squads) to one of the three interventions. This cluster randomization was effective because there were no significant differences between groups in these two covariates. Consequently, they and their interactions with experimental factors were not included in statistical models to avoid overparameterization.”*

**Comment: Biological maturation**

**The inclusion of U13–U17 players introduces substantial variability in biological maturation. Although maturity offset is reported, it does not appear to be included as a covariate in the analyses.**

**Please clarify:**

**• Was maturation statistically controlled?**

**• Could observed changes reflect developmental processes rather than intervention effects?**

**Given the age range, maturation is a relevant potential confounder.**

Response: Thank you for highlighting this aspect. The comparison of the maturity offset values between groups yielded no statistically significant differences (*p* = 0.674). Therefore, this factor was not included in the statistical analyses. For clarification, we have added the following information to the Material and Methods section (cf. Participants): *“To guard against potential confounding effects of between-subject differences in chronological age, maturity offset, and intervention type, we randomly assigned groups of 6–8 players per age group (i.e., U13, U15, U17 squads) to one of the three interventions. This cluster randomization was effective because there were no significant differences between groups in these two covariates. Consequently, they and their interactions with experimental factors were not included in statistical models to avoid overparameterization.”*

**Comment: Task specificity and transfer**

**Balance training adaptations are often task-specific with limited transfer between tasks.**

**The manuscript would benefit from elaboration on:**

**• Why improvements in YBT-LQ and SLDL are expected to generalize to soccer performance or injury prevention.**

**• The ecological validity of these assessments in elite youth soccer.**

**Addressing specificity versus transfer would strengthen the interpretation.**

Response: We agree with the reviewer that adaptations related to balance training are often task-specific with limited transfer between tasks. However, we did not examine improvements in YBT-LQ and SLDL—which were the focus of the previous study [1]—but rather examined improvements in unipedal stance with and without leg swing (see Figure 2 A–B). From a practical perspective, the unipedal stance is a typical task in soccer, such as when kicking, crossing, and passing the ball. From a theoretical perspective, we have followed the conceptual framework developed by Paillard [10], who distinguishes between *ecological* (i.e., contextualized, specific conditions related to the practiced type of sport) and *non-ecological* (i.e., decontextualized, unspecific conditions unrelated to the practiced type of sport) *test conditions*. Based on this distinction, the unipedal stance can be classified as ecologically valid for the assessment of soccer players. We have added this information to the Material and Methods section (cf. Assessment of balance performance): *“According to the conceptual framework of Paillard [10], the unipedal stance represents a contextualized, specific test condition—such as during kicking, crossing, and passing a soccer ball—related to the practiced type of sport (i.e., soccer) and is therefore suitable for an ecologically valid assessment of soccer players.”*

**Comment: Discussion and Conclusion: Conceptual Contribution and Tone**

**While the study is well conducted and addresses a relevant applied question, its conceptual contribution appears primarily incremental rather than novel. The findings confirm that unilateral balance-based training can modulate interlimb asymmetry — an effect that has been previously reported in other populations and settings. The present study adds valuable applied evidence from an elite youth soccer context and compares two unilateral training modalities within the same cohort. However, it does not establish a fundamentally new theoretical framework regarding asymmetry, neuromuscular adaptation, or injury prevention. For this reason, I recommend that the Discussion and Conclusion be written in a more conservative tone.**

**Specifically:**

**• Avoid overstating novelty.**

**• Avoid strong injury-prevention implications, as injury reduction was not directly demonstrated.**

**• Position the contribution as applied extension rather than conceptual advancement.**

**• Clearly distinguish between statistical findings and broader performance or injury claims.**

**Reframing the conclusions as incremental applied evidence would improve scientific balance and credibility.**

Response: Based on your suggestions, we have revised the Discussion and Conclusions section. Specifically, we have now avoided emphasizing the novelty of the study. In addition, we have avoided drawing implications regarding injury prevention. The present study was also presented as a contribution to the practical application of previous findings rather than as a conceptual advancement. Furthermore, a clearer distinction was made between statistical results and more general statements regarding performance or injuries.

Finally, the Conclusions section was rephrased to reflect incremental applied evidence.

The following was added to the Discussion section: *“Thus, the present study represents an applied extension of a previous investigation [1] on the effects of single-mode BT versus combined BT+PT on proactive and reactive balance performance as well as muscle activity in young soccer players.”*

The following was deleted from the Discussion section: *“~~An improvement in interlimb symmetry, or a reduction in interlimb asymmetry, is noteworthy and suggests that training interventions can positively influence this important indicator associated with detrimental effects on physical and sports performance [3, 4] and an increased risk for time loss non-contact lower limb injuries [1, 2].~~”*

The following was changed in the Conclusions section: *“The present study adds further applied evidence to previous investigations of training-related changes on interlimb asymmetry.”*

**Comment: Title**

**The current title (“Role of training”) is rather broad and does not adequately reflect:**

**• the unilateral intervention design,**

**• the comparative training modalities,**

**• and the static vs. dynamic postural control focus.**

**A more precise title would better represent the study and clearly differentiate it from related previously published work.**

Response: We agree with the reviewer and have created a more precise title which reads as follows: *Effects of unilateral single-mode balance training compared to combined balance and plyometric training on soccer players’ interlimb asymmetry in static and dynamic balance performance*

**Specific Comments**

**Abstract**

**• Strengthen the applied relevance (injury prevention and/or performance context).**

**• Clarify why comparing single-mode and combined training modalities is important.**

**• Specify playing level (elite academy).**

**• Indicate whether the intervention was conducted in-season.**

**• Elaborate briefly on the practical meaning of the observed improvements.**

Response: In accordance with your suggestions, we have indicated that the performance context represents the applied relevance of the present study. Further, we have clarified that complementary adaptations are assumed for the combined training modality. Information about the playing level (Tier level 3) has been added (as well as in the Material and Method section, cf. Participants). We have indicated that the intervention was conducted in-season. In terms of practical meaning, we think the results of the in-depth analyses are particularly relevant. These are CoP-based indices that provide insight into the regulatory processes underlying balance performance. We have therefore added the following statement: *„From a practical perspective, the improvements in CoP-based indices suggest that processes of balance regulation in particular benefit from the combined BT+PT.“*

**Comment: Introduction**

**• Provide sport-specific evidence for youth soccer rather than relying predominantly on basketball data.**

**• Clarify whether clinically meaningful asymmetries are documented in elite youth soccer.**

**• Discuss adaptive vs. maladaptive asymmetry.**

**• Address whether balance adaptations persist under fatigue conditions.**

Response: We agree with the reviewer and have now included studies including soccer players [6] and reported findings from studies that investigated different sports including soccer [9]. Further, clinically meaningful asymmetries are documented in athletes (including soccer players) [11]. Moreover, there is evidence that interlimb asymmetry increases following fatigue [12]. All these aspects are now included in the Introduction section: *“Specifically, Smith et al. [9] evaluated balance performance in collegiate athletes from different sports (e.g., soccer) and tracked lower-limb injuries over the course of a season. Of the 184 participants, 81 sustained non-contact injuries. The authors also found that athletes with a greater than 4 cm difference in anterior reach between the left and right legs had a 2.2-fold higher injury risk. There is further evidence from a performance-related perspective showing that interlimb asymmetry in postural control is related to competition level in soccer players [6] which could result in worse athletic performance [13]. Precisely, Gonzalez-Fernandez et al. [6] reported that greater interlimb asymmetry in balance performance depends on the competition level, with the largest values observed among amateur soccer players (5.3–12.0%), followed by semi-professional players (4.0–9.0%), and professional players (1.7–4.3%). For athletes including soccer players, interlimb asymmetry values greater than 10% are considered maladaptive and clinically meaningful [2,3,11]. Furthermore, there is evidence that asymmetry increases following fatigue [12], which can certainly occur in soccer due to long or intense periods of training/competition.”*

**Comment: Methods**

**• Clarify details of cluster randomization.**

**• Clarify whether sitting height was measured.**

**• Specify how maturation was balanced and handled statistically.**

Response: As already mentioned in response to one of your previous comments, groups of 6–8 players per age group (squad) were randomly assigned to one of the three interventions. This approach was used to ensure an even distribution of players across age categories and thus to rule out the influence of chronological age. Consequently, there was no further need to account for cluster randomization in the statistical modelling. For clarification, we have added the following information to the Material and Methods section (cf. Participants): *“To guard against potential confounding effects of between-subject differences in chronological age, maturity offset, and intervention type, we randomly assigned groups of 6–8 players per age group (i.e., U13, U15, U17 squads) to one of the three interventions. This cluster randomization was effective because there were no significant differences between groups in these two covariates. Consequently, they and their interactions with experimental factors were not included in statistical models to avoid overparameterization.”*

Standing and sitting height were measured and used to calculate maturity offset using the regression equation provided by Mirwald et al. [49] that comprise measures of chronological age, body mass, standing and sitting height. More precisely, maturity offset = -9.236 + (0.0002708 × leg length × sitting height) – (0.001663 × age × leg length) + (0.007216 × age × sitting height) + (0.02292 × body mass / standing height). This information has been added to the Material and Methods section (cf. Assessments of anthropometric variables): *“Maturity offset was calculated as years from PHV by using the regression equation provided by Mirwald et al. [14].”*

In terms of maturation, the comparison of the maturity offset values between groups yielded no statistically significant differences. Therefore, this factor was not included in the statistical analyses. For clarification, we have added the following information to the Material and Methods section (cf. Participants): *“To guard against potential confounding effects of between-subject differences in chronological age, maturity offset, and intervention type, we randomly assigned groups of 6–8 players per age group (i.e., U13, U15, U17 squads) to one of the three interventions. This cluster randomization was effective because there were no significant differences between groups in these two covariates. Consequently, they and their interactions with experimental factors were not included in statistical models to avoid overparameterization.”*

**Comment: Intervention**

**• Clarify whether plyometric exercises were performed unilaterally or bilaterally.**

**• Specify take-off and landing leg.**

**• Ensure key unilateral aspects are clearly stated in the abstract.**

**• Provide sufficient detail for reproducibility.**

Response: For clarification, we have added the information that the plyometric exercises in the unilateral combined BT+PT group were performed with the non-dominant leg used for take-off and landing (cf. Material and Methods section, Interventions). In the Abstract, we have now specified that the non-dominant leg was used during training.

**Comment: Minor Points**

**• Consider adjustment for multiple comparisons.**

**• Ensure consistent terminology regarding asymmetry thresholds.**

Response: The experimental design implies a priori theoretically expected higher-order interactions between contrasts. In this case, the test statistics associated with the respective contrasts replace sequences of significant omnibus *F*-tests followed by multiple comparisons *comparisons [15,16].* For clarification, we have added the following information to the Material and Methods section (cf. Inferential statistics): *We estimated fixed effects for the two contrasts defined for control and training groups, pretest/posttest, static/dynamic balance condition, task difficulty level, and dominant/non- dominant leg. The first contrast (C1) tested the average of the two training groups against the active CON group; the second contrast (C2) tested the difference between the two training groups. Contrasts with effect coding were used for the other factors. We also included all simple interactions between these main effects and six theoretically relevant three-factor interactions arising from the combination of time × (C1 | C2) × (balance condition | task difficulty level leg). These contrasts and associated interactions were dictated by the design and specified* a priori *with a clear expectation about the profile of means, not as omnibus F-tests to be followed up with post-hoc t-tests adjusted for multiple comparisons [15,16]. The design was used both for the GLMM for the parameter success rate and the LMM for the CoP-based parameters. For unbalanced designs, (G)LMMs preserve statistical power better than repeated-measures ANOVAs.*

Further, we have proofread the whole manuscript and confirm that terminology regarding asymmetry thresholds is now consistent, i.e., a limb symmetry index value above 90% reflects interlimb symmetry in postural control, whereas values below this threshold indicate interlimb asymmetry.

**Comment: Final Recommendation**

**The manuscript addresses an interesting and applied question within elite youth soccer and is based on a valuable dataset collected in a real-world setting. However, conceptual clarification, methodological transparency, and more conservative interpretation are required before publication. For these reasons, I recommend major revision.**

Response: We thank the reviewer for providing constructive criticism. Your comments helped us to focus our findings and improve the manuscript. We hope that the revised manuscript meets the requirements and is now suitable for publication in PLoS One.

**Responses to Reviewer #2:**

**Comment: Thank you for the opportunity to review this manuscript. The study addresses a relevant topic with a considerable sample size, and the randomized design is appreciated. However, I have several concerns regarding the conceptual framing and the statistical approach. Please see specific comments below:**

Response: We thank the reviewer for this affirmative comment. Further, we are grateful for the time and effort invested in providing constructive criticism. Below you will find the responses to your comments. All changes made in response to your comments were highlighted in grey in the revised manuscript.

**Comment: Abstract**

**The conclusion reads that the combined BT+PT is “presumably more suitable”. However, the results show that BT is superior for success rate (although they performed significantly worse during pre), BT+PT is superior for CoP, and that there are no differences for LSI. Thus, this does not seem fully consistent with the conclusion. Therefore, I would recommend toning this down and presenting a more balanced interpretation.**

Response: We agree with the reviewer. We think the results of the in-depth analyses are particularly relevant. These are CoP-based indices that provide insight into the regulatory processes underlying balance performance. We have therefore changed the statement as follows: *”Given the greater improvements in parameters obtained from the more in-depth analyses (i.e., CoP-based indices) in favor of the combined BT+PT, this training modality seems to be more suitable for enhancing interlimb postural control.“*

**Comment: Methods/Results**

**L102: Participants were allocated to the training groups using cluster randomization across teams. I wonder, why you did not account for or adjust for the clustering approach in your statistical model, as this might violate independence assumptions and thus inflate your results?**

Response: We apologize for lack of precision. Players came from three age groups (squads) from one team (i.e., SG Dynamo Dresden e.V.). With “cluster randomization” we referred to the random assignment of groups of 6–8 players per age group (squad) to one of the three interventions. This approach was used to ensure an even distribution of players across age categories and thus to rule out the influence of chronological age. Thus, neither team nor age group nor randomization cluster could be included as a random factor in the (G)LMMs; they would have only 1, 3, and levels, respectively. For clarification, we added the following information to the Material and Methods section (cf. Participants): *“To guard against potential confounding effects of between-subject differences in chronological age, maturity offset, and intervention type, we randomly assigned groups of 6–8 players per age group (i.e., U13, U15, U17 squads) to one of the three interventions. This cluster randomization was effective because there were no significant differences between groups in these two covariates. Consequently, they and their interactions with experimental factors were not included in statistical models to avoid overparameterization.”*

**L144: The control group performed stretching exercises including the lower body. However, stretching may also influence proprioception and balance; especially when performed at a single-legged stance. Thus, the control condition might have been not entirely neutral.**

Response: We agree with the reviewer that chronic stretching can lead to changes in the tension tolerance of muscles and connective tissue [17] but has only small to moderate effects on static and dynamic balance parameters [18]. Therefore, beneficial effects on proprioception and balance cannot be completely excluded. Taking this criticism into account, we have added a limitations section stating (cf. Discussion): *“Fourth, the intervention program completed by the active control group might have been not entirely neutral since stretching exercises can also have beneficial effects on proprioception and balance performance [18].”*

**L152: Participants were exhibited to a high amount of regular training (soccer + athletic + PE), which might also have included elements comparable to the additional intervention. Thus, it might be hard to isolate the contribution of the relatively low amount of additional training of the intervention (2x 30 min/w)?**

Response: The amount of regular training (i.e., soccer-specific training of 360–420 minutes per week and athletic training of 90–180 minutes per week) was equally distributed between groups. Consequently, the detected differences in static and dynamic balance outcomes (see Results) are related to the specificity of the respective training modality (i.e., unilateral single-mode BT group *vs.* unilateral combined BT+PT group *vs.* active control group).

**L176: Six seconds seems to be rather short? Could you provide reliability data for this test?**

Response: We agree with the reviewer that a 6-s measurement duration for the dynamic balance test condition seems to be rather short but are in agreement with previous studies [19,20] and was designed to minimize fatigue. However, reliability data obtained from the three data-collection trials (at pretesting) of the present study revealed the following findings (cf. Material and Methods section, Assessment of balance performance): *“Reliability data were calculated from the present study and yielded for the assessment of static balance ICC values (95% CI) of 0.50 (0.34, 0.65) for ap-COP RMS (dominant leg), 0.60 (0.46, 0.73) for ap-COP RMS (non-dominant leg), 0.55 (0.40, 0.68) for ml-COP RMS (dominant leg), and 0.60 (0.45, 0.72) for ml-COP RMS (non-dominant leg), indicating “fair to good” reliability [21]. For dynamic balance, ICC values (95% CI) amounted to 0.50 (0.35, 0.65) for ap-COP RMS (dominant leg), 0.47 (0.31, 0.62) for ap-COP RMS (non-dominant leg), 0.41 (0.24, 0.58) for ml-COP RMS (dominant leg), and 0.52 (0.36, 0.66) for ml-COP RMS (non-dominant leg), also indicating “fair to good” reliability.”*

**L181: The adaptive difficulty approach is interesting, but as difficulty is increased individually in relation to the participants performance level, participants are effectively tested under different conditions, which may reduce comparability between subjects. Could you clarify how this affects interpretability of group differences?**

Response: We apologize for being imprecise. The incremental increase in task difficulty from level 3 over levels 4 and 5 to level 6 was not individualized but was identical for all players. However, the number of players who successfully completed level 3, or levels 3 and 4, or levels 3 to 5, or levels 3 to 6 differed. Since the subsequent statistical analyses were conducted per level (**see Figure 3 C, 4 B, and 5 B, D**), the comparability between players is fulfilled. For clarification, we have changed the wording in the Material and Methods section (cf. Assessment of balance performance) as follows: *“Consequently, the task difficulty was adjusted incrementally for all players starting from level 3 over levels 4 and 5 to level 6. Using this procedure, the highest difficulty level that could be successfully achieved with both the dominant and non-dominant leg was determined for each player.”*

**L190: Was there a familiarization session prior to data collection? Moreover, you state that a trial was discarded and repeated if participants failed. What was the min and max number of trials per participants? Moreover, later in L210/211 you mention that the maximum number of valid trials was 3 and that the LSI was computed as min/max. Does that mean that for some participants you used a lower number than 3 trials to determine min and max?**

Response: There was no familiarization session prior to data collection, but two practice trials were performed to familiarize with the test respective condition followed by three data-collection trials. For clarification, we have changed the wording in the Material and Methods section (cf. Assessment of balance performance) as follows: *“For each difficulty level, two practice trials were performed to familiarize with the respective test condition followed by three data-collection trials, and the mean of the data-collection trials was used for further analyses.”*

Further, the minimal number of valid trials per player was one and the maximal number of valid trials per player was three. Consequently, for some players, fewer than three trials were used to determine the minimum and maximum values. One of the advantages of (G)LMMs is that they preserve statistical power better than traditional repeated-measures ANOVA when faced with missing values; they also take the differences in number of observations into account in estimation of fixed effects and variance components. The full random-effects model (RES) with variance components (VCs) and correlation parameters (CPs) for grand mean (GM), times, balance conditions, difficulty levels, and legs was supported by the data (**see S1 Table**).

**L195: Is r = 0.57 really an indicator for sufficient validity (~ 32% of variance explained)? Also, the criterion (against what was the UST tested?) should be specified. Moreover, the ICC should be reported and interpreted by including its 95%CI.**

Response: The cited *r*-value of 0.57 refers to a study conducted with late midlife women with an age range of 58–69 years that is quite different from the present one (age range: 12–16 years). In another study by Goldie et al. [22], young adults (mean age: 28.1 ± 8.0 years) were examined. Compared to the step stance, the variance explained amounted to 58.5 (preferred leg) and 65.0% (non-preferred leg) for the static test condition and 63.2 (preferred leg) and 79.0% (non-preferred leg) for the dynamic test condition. Consequently, we have now cited the study of Goldie et al. [22] instead of Clark [23] (cf. Material and Methods section, Assessment of balance performance): *“The unipedal stance test is considered a valid measure of balance performance [22]. Compared to the step stance, the variance explained amounted to 58.5% (preferred leg) and 65.0% (non-preferred leg) for the static test condition and 63.2% (preferred leg) and 79.0% (non-preferred leg) for the dynamic test condition.”*

Further, the study of Clark [23] does not provide information about the 95% CI. Thus, we now have calculated and reported *ICC*-values with 95% CI from the three data-collection trials (at pretesting) of the present study (cf. Material and Methods section, Assessment of balance performance): *“Reliability data were calculated from the present study and yielded for the assessment of static balance ICC values (95% CI) of 0.50 (0.34, 0.65) for ap-COP RMS (dominant leg), 0.60 (0.46, 0.73) for ap-COP RMS (non-dominant leg), 0.55 (0.40, 0.68) for ml-COP RMS (dominant leg), and 0.60 (0.45, 0.72) for ml-COP RMS (non-dominant leg), indicating “fair to good” reliability [21]. For dynamic balance, ICC values (95% CI) amounted 0.50 (0.35, 0.65) for ap-COP RMS (dominant leg), 0.47 (0.31, 0.62) for ap-COP RMS (non-dominant leg), 0.41 (0.24, 0.58) for ml-COP RMS (dominant leg), and 0.52 (0.36, 0.66) for ml-COP RMS (non-dominant leg), also indicating “fair to good” reliability.”*

**L252: While I understand the argument for restricting the analysis for the success rate to levels 5-6 and the CoP to levels 3-4, this seems to be a post-hoc decision based on the distribution of your data, rather than a priori decision. Moreover, this results in different datasets for different outcomes and makes it more difficult to compare between the different outcomes.**

Response: We agree with the reviewer that using levels 5 and 6 for the parameters success rate and levels 3 and 4 for the CoP-based parameters was a post-hoc decision rather than a priori decision. However, this was not based on the distribution of our data, rather than to avoid ceiling effects of levels 3 and 4 for the parameter success rate and to avoid bias due to selective drop-out (floor effects) of level-5 and level-6 trials from less-balanced players for CoP-based parameters (cf. Results, Preprocessing and overview). This resulted in different datasets for different outcomes. Consequently, we did not compare our findings between the different outcomes but used a three-step analysis (first step: binary parameters; second step: CoP-based indices; third step: LSI indices). The discussion of the results also follows this structure. When drawing conclusions, we believe that the results of the more sophisticated, in-depth analysis (i.e., CoP-based indices) are particularly relevant. We have therefore focused on these results in formulating our conclusions.

(cf. Abstract): *„Given the greater improvements in parameters obtained from the more in-depth analyses (i.e., CoP-based indices) in favor of the combined BT+PT, this training modality seems to be more suitable for enhancing interlimb postural control.“*

(cf. Conclusions): *„The greater effectiveness of combined BT+PT in parameters resulting from more sophisticated, in-depth analyses suggest that this training modality is probably better suited to positively influencing interlimb postural control.“*

**L228: The model includes multiple main effects and several interaction terms (including 3-way interactions) with a comparably large sample size for sport science papers, but still relatively small sample size of n = 57). This seems quite complex and raises the concern of the models being overfitted. Are all these interactions theoretically justified? This is especially relevant as some findings are based on bivariate classification of results into significant/non-significant around p-values close to the traditional 0.05-threshold in complex interaction terms. More importantly, many of the key conclusions rely on higher-order interactions. For example, effects such as time x group x condition x difficulty level x leg (implicitly decomposed across multiple 3-way terms) are very difficult to interpret. From a physiological perspective, I’m not convinced that such interaction structures are meaningful? This should at least be discussed critically. Moreover, you analyze multiple dependent variables, sometimes after computing the dependent variable as a ratio. I feel that it would strengthen the paper if there would be a clearly defined primary outcome.**

Response: We agree with the reviewer that the experiment implemented a complex design. The three-factor interactions were expected for observed values (i.e., the basic group x time interaction for any intervention design was expected to be moderated by at least one of the other design factors). Therefore, we opted to specify the expected effects as *a priori* contrasts (rather than following up significant omnibus *F*-tests with multiple comparisons; see Hays [16] and Schad et al. [15]. For clarification, we have added the following information to the Material and Methods section (cf. Inferential statistics): “*We estimated fixed effects for the two contrasts defined for control and training groups, pretest/posttest, static/dynamic balance condition, task difficulty level, and dominant/non- dominant leg. The first contrast (C1) tested the average of the two training groups against the active CON group; the second contrast (C2) tested the difference between the two training groups. Contrasts with effect coding were used for the other factors. We also included all simple interactions between these main effects and six theoretically relevant three-factor interactions arising from the combination of time × (C1 | C2) × (balance condition | task difficulty level leg). These contrasts and associated interactions were dictated by the design and specified* a priori *with a clear expectation about the profile of means, not as omnibus F-tests to be followed up with post-hoc t-tests adjusted for multiple comparisons [15,16]. The design was used both for the GLMM for the parameter success rate and the LMM for the CoP-based parameters. For unbalanced designs, (G)LMMs preserve statistical power better than repeated-measures ANOVAs.”*

The reviewer also raises a question about “multiple dependent variables, sometimes after computing the dependent variable as a ratio.” In response, we have added the following information to the Material and Methods section (cf. Inferential statistics): “*While contrast specifications for all (G)LMMs were determined by the experimental design, the choice of measures was informed by the outcomes. Given respective ceiling and floor effects, the first decision was to analyze success rate only for two difficult conditions (levels 5 and 6) and CoP measures only for two easy conditions (levels 3 and 4). Aside from bypassing the interpretational problems, we reduced dependency with analyses of two non-overlapping subsets of the observations. As far as ap- and ml-CoP-measures were concerned, a distributional analysis clearly established the need for log-transformation to meet assumptions of normal distribution for model residuals. These are the primary dependent variables in this field, but for the present context, ap-CoP was expected to relate more directly to the theoretical issue of leg asymmetry (i.e., forward/backward sway). Thus, success rate and ap-CoP assessed with non-overlapping subsets of data were our primary choices. Further, we anticipated an interest in additional CoP analyses. Obviously, ml-CoP was to be checked as a complementary follow-up. Indeed, this analysis revealed some unexpected results that need to be followed up. Moreover, leg asymmetry is usually operationalized as a ratio between the two legs. Given that we analyzed log(CoP) values, the leg main effect was estimated as a ratio in the original metric. To align our analysis with the established practice of the field, we included a post-hoc LMM using this ratio directly as dependent variable. This analysis was carried out both for ratios of ap- and ml-CoP values. Finally, again to align our study with past practice, we also computed and analyzed leg asymmetry based on the established formula 1 for both ap- and ml-CoP.”*

**Comment: Table 2: There is a typo (BP+PT instead of BT+PT)**

Response: The typo was corrected.

References used for revision

1.	Muehlbauer, T.; Borgmann, K.; Limpach, S.; Krombholz, D.; Panzer, S. Impact of unilateral single-mode balance training vs. combined balance and plyometric training on soccer players' interlimb asymmetry in balance and neuromuscular performance. *Front Sports Act Living* **2026**, *8*, 1743152, doi:10.3389/fspor.2026.1743152.

2.	Grindem, H.; Logerstedt, D.; Eitzen, I.; Moksnes, H.; Axe, M.J.; Snyder-Mackler, L.; Engebretsen, L.; Risberg, M.A. Single-legged hop tests as predictors of self-reported knee function in nonoperatively treated individuals with anterior cruciate ligament injury. *Am J Sports Med* **2011**, *39*, 2347-2354, doi:10.1177/0363546511417085.

3.	Logerstedt, D.; Grindem, H.; Lynch, A.; Eitzen, I.; Engebretsen, L.; Risberg, M.A.; Axe, M.J.; Snyder-Mackler, L. Single-legged hop tests as predictors of self-reported knee function after anterior cruciate ligament reconstruction: the Delaware-Oslo ACL cohort study. *Am J Sports Med* **2012**, *40*, 2348-2356, doi:10.1177/0363546512457551.

4.	Paillard, T. Plasticity of the postural function to sport and/or motor experience. *Neurosci Biobehav Rev* **2017**, *72*, 129-152, doi:10.1016/j.neubiorev.2016.11.015.

5.	Maloney, S.J. The relationship between asymmetry and athletic performance: a critical review. *J Strength Cond Res* **2019**, *33*, 2579-2593, doi:10.1519/JSC.0000000000002608.

6.	Gonzalez-Fernandez, F.T.; Martinez-Aranda, L.M.; Falces-Prieto, M.; Nobari, H.; Clemente, F.M. Exploring the Y-Balance-Test scores and inter-limb asymmetry in soccer players: differences between competitive level and field positions. *BMC Sports Sci Med Rehabil* **2022**, *14*, 45, doi:10.1186/s13102-022-00438-w.

7.	Rasool, J.; George, K. The impact of single-leg dynamic balance training on dynamic stability. *Physical Therapy in Sport* **2007**, *8*, 177-184.

8.	Oliveira, A.S.; Brito Silva, P.; Farina, D.; Kersting, U.G. Unilateral balance training enhances neuromuscular reactions to perturbations in the trained and contralateral limb. *Gait Posture* **2013**, *38*, 894-899, doi:10.1016/j.gaitpost.2013.04.015.

9.	Smith, C.A.; Chimera, N.J.; Warren, M. Association of y balance test reach asymmetry and injury in division I athletes. *Med Sci Sports Exerc* **2015**, *47*, 136-141, doi:10.1249/MSS.0000000000000380.

10.	Paillard, T. Relationship between sport expertise and postural skills. *Front Psychol* **2019**, *10*, 1428, doi:10.3389/fpsyg.2019.01428.

11.	Gokeler, A.; Welling, W.; Benjaminse, A.; Lemmink, K.; Seil, R.; Zaffagnini, S. A critical analysis of limb symmetry indices of hop tests in athletes after anterior cruciate ligament reconstruction: A case control study. *Orthop Traumatol Surg Res* **2017**, *103*, 947-951, doi:10.1016/j.otsr.2017.02.015.

12.	Penedo, T.; Polastri, P.F.; Rodrigues, S.T.; Santinelli, F.B.; Costa, E.C.; Imaizumi, L.F.I.; Barbieri, R.A.; Barbieri, F.A. Motor strategy during postural control is not muscle fatigue joint-dependent, but muscle fatigue increases postural asymmetry. *PLoS One* **2021**, *16*, e0247395, doi:10.1371/journal.pone.0247395.

13.	Bishop, C.; Turner, A.; Read, P. Effects of inter-limb asymmetries on physical and sports performance: a systematic review. *J Sports Sci* **2018**, *36*, 1135-1144, doi:10.1080/02640414.2017.1361894.

14.	Mirwald, R.L.; Baxter-Jones, A.D.; Bailey, D.A.; Beunen, G.P. An assessment of maturity from anthropometric measurements. *Med Sci Sports Exerc* **2002**, *34*, 689-694.

15.	Schad, D.; Hohenstein, S.; Vasishth, S.; Kliegl, R. How to capitalize on a priori contrasts in linear (mixed) models: A tutorial. *J Memory Language* **2020**, *110*, 104038.

16.	Hays, W.L. *Statistics for the social sciences*; Holt, Rinehart & Winston of Canada Ltd.: New York, 1973.

17.	Rubini, E.C.; Costa, A.L.; Gomes, P.S. The effects of stretching on strength performance. *Sports Med* **2007**, *37*, 213-224.

18.	Lohmann, L.H.; Zech, A.; Ploschberger, G.; Oraze, M.; Jochum, D.; Warneke, K. Acute and chronic effects of stretching on balance: a systematic review with multilevel meta-analysis. *Front Med (Lausanne)* **2024**, *11*, 1451180, doi:10.3389/fmed.2024.1451180.

19.	Leinen, P.; Muehlbauer, T.; Panzer, S. Single-leg balance performance in sub-elite young soccer players and swimmers as a function of age and sports experience. *Journal of Motor Learning and Development* **2019**, *7*, 374-388.

20.	Teixeira, L.A.; de Oliveira, D.L.; Romano, R.G.; Correa, S.C. Leg preference and interlateral asymmetry of balance stability in soccer players. *Res Q Exerc Sport* **2011**, *82*, 21-27.

21.	Fleiss, J.L. *Reliability of measurement. The design and analysis of clinical experiments.*; Wiley: New York, 1986.

22.	Goldie, P.A.; Bach, T.M.; Evans, O.M. Force platform measures for evaluating postural control: reliability and validity. *Arch Phys Med Rehabil* **1989**, *70*, 510-517.

23.	Clark, M.S. The unilateral forefoot balance test: reliability and validity for measuring balance in late midlife women. *Journal of Physiotherapy* **2007**, *35*, 110-118.
