# Supplementary material for: Effects of unilateral single-mode balance training compared to combined balance and plyometric training on soccer players’ interlimb asymmetry in static and dynamic balance performance
Source: PLoS One. 2026 Sep 16;21(9):e0358565. doi: 10.1371/journal.pone.0358565 (PMC13581024; doi:10.1371/journal.pone.0358565)
Supplement: Supplementary file 5 [file pone.0358565.s006.docx]

Manuscript Number: **PONE-D-26-01855R1**

Title: **Effects of unilateral single-mode balance training compared to combined balance and plyometric training on soccer players’ interlimb asymmetry in static and dynamic balance performance**

**Responses to Reviewer #2:**

**COMMENT: I highly appreciate your revised manuscript! Although the manuscript improved, I have some further comments regarding the responses to my initial points. Please see specific comments below:**

Response: We thank the reviewer for this affirmative comment. Further, we are grateful for the time and effort invested in providing constructive criticism. Below you will find the responses to your comments. All changes made in response to your comments were highlighted in grey in the revised manuscript.

**COMMENT on the Abstract – The conclusion reads that the combined BT+PT is “presumably more suitable”. However, the results show that BT is superior for success rate (although they performed significantly worse during pre), BT+PT is superior for CoP, and that there are no differences for LSI. Thus, this does not seem fully consistent with the conclusion. Therefore, I would recommend toning this down and presenting a more balanced interpretation.**

**Response: We agree with the reviewer. We think the results of the in-depth analyses are particularly relevant. These are CoP-based indices that provide insight into the regulatory processes underlying balance performance. We have therefore changed the statement as follows: ”Given the greater improvements in parameters obtained from the more in-depth analyses (i.e., CoP-based indices) in favor of the combined BT+PT, this training modality seems to be more suitable for enhancing interlimb postural control.“**

**Reply: Even though this is now clearer and toned down in wording, I still feel that the manuscript selectively prioritizes the CoP-based outcomes over the other outcome domains. While I agree that CoP-based measures may provide a more detailed assessment of postural regulation, this does not necessarily make them more meaningful or more valid than the other outcomes. Importantly, superiority of BT+PT is still mainly concluded based on the outcome domain favoring this intervention, whereas the binary outcome favored BT and LSI outcomes showed largely no differences. Thus, I would still recommend presenting the findings in a more balanced manner and emphasizing the inconsistency across outcome domains more explicitly.**

Response: We agree with the reviewer and changed the statement as follows: *”Due to the inconsistency across outcome domains, it cannot be concluded that one training mode is superior to the other. However, the more in-depth analysis (i.e., CoP-based indices) yields findings in favor of the combined BT+PT and suggests that regulatory processes, in particular, benefit from this training modality.“*

**COMMENT on L152: Participants were exhibited to a high amount of regular training (soccer + athletic + PE), which might also have included elements comparable to the additional intervention. Thus, it might be hard to isolate the contribution of the relatively low amount of additional training of the intervention (2x 30 min/w)?**

**Response: The amount of regular training (i.e., soccer-specific training of 360–420 minutes per week and athletic training of 90–180 minutes per week) was equally distributed between groups. Consequently, the detected differences in static and dynamic balance outcomes (see Results) are related to the specificity of the respective training modality (i.e., unilateral single-mode BT group vs. unilateral combined BT+PT group vs. active control group).**

**Reply: I do not think my concern was fully addressed. My point was less about equal distribution between groups, but rather about the interpretability of relatively small intervention effects in the context of a very high overall training volume. Even if background training was comparable between groups, it still remains difficult to isolate the contribution of the additional intervention. I still feel this is a considerable limitation.**

Response: We agree with the reviewer and have added this aspect to the limitations paragraph (cf. Discussion): *“Eighth, although the total training volume was distributed equally among the groups, the proportion of the additional intervention (i.e., 60 minutes per week) was smaller than the proportion of soccer-specific training (360–420 minutes per week) and athletic training (90–180 minutes per week). Therefore, it cannot be ruled out that the observed effects were caused solely by the intervention.”*

**COMMENT on L176: Six seconds seems to be rather short? Could you provide reliability data for this test?**

**Response: We agree with the reviewer that a 6-s measurement duration for the dynamic balance test condition seems to be rather short but it is in agreement with previous studies [19,20] and was designed to minimize fatigue. However, reliability data obtained from the three data-collection trials (at pretesting) of the present study revealed the following findings (cf. Material and Methods section, Assessment of balance performance): “Reliability data were calculated from the present study and yielded for the assessment of static balance ICC values (95% CI) of 0.50 (0.34, 0.65) for ap-COP RMS (dominant leg), 0.60 (0.46, 0.73) for ap-COP RMS (nondominant leg), 0.55 (0.40, 0.68) for ml-COP RMS (dominant leg), and 0.60 (0.45, 0.72) for mlCOP RMS (non-dominant leg), indicating “fair to good” reliability [21]. For dynamic balance, ICC values (95% CI) amounted to 0.50 (0.35, 0.65) for ap-COP RMS (dominant leg), 0.47 (0.31, 0.62) for ap-COP RMS (non-dominant leg), 0.41 (0.24, 0.58) for ml-COP RMS (dominant leg), and 0.52 (0.36, 0.66) for ml-COP RMS (non-dominant leg), also indicating “fair to good” reliability.”**

**Reply: Thank you for adding the reliability analyses. However, I would argue that ICC values in the range of 0.4 to 0.6 are only moderate at best, particularly given the complexity of the subsequent interaction analyses. This should, in my opinion, be discussed more critically. Moreover, low ICC values inherently imply a substantial measurement error. For example, an ICC of 0.50 corresponds roughly to a SEM of 0.7 SD and an MDC95 of almost 2 SD. Thus, the relatively small observed differences and especially complex interaction effects should be interpreted considerably more cautiously.**

Response: The other reviewer also raised this issue. Consequently, we have changed “fair to good” to “poor to moderate” reliability. In addition, we have added this aspect to the limitations paragraph (cf. Discussion): *“Sixth, between-trial test-retest reliability was poor to moderate for the dynamic balance task. Therefore, it cannot be ruled out that the low repeatability of data collection may have affected the accuracy of the present findings.”*

**COMMENT on L252: While I understand the argument for restricting the analysis for the success rate to levels 5-6 and the CoP to levels 3-4, this seems to be a post-hoc decision based on the distribution of your data, rather than a priori decision. Moreover, this results in different datasets for different outcomes and makes it more difficult to compare between the different outcomes.**

**Response: We agree with the reviewer that using levels 5 and 6 for the parameters success rate and levels 3 and 4 for the CoP-based parameters was a post-hoc decision rather than a priori decision. However, this was not based on the distribution of our data, rather than to avoid ceiling effects of levels 3 and 4 for the parameter success rate and to avoid bias due to selective drop-out (floor effects) of level-5 and level-6 trials from less-balanced players for CoP-based parameters (cf. Results, Preprocessing and overview). This resulted in different datasets for different outcomes. Consequently, we did not compare our findings between the different outcomes but used a three-step analysis (first step: binary parameters; second step: CoP-based indices; third step: LSI indices). The discussion of the results also follows this structure. When drawing conclusions, we believe that the results of the more sophisticated, in-depth analysis (i.e., CoP-based indices) are particularly relevant. We have therefore focused on these results in formulating our conclusions.**

**(cf. Abstract): „Given the greater improvements in parameters obtained from the more in-depth analyses (i.e., CoP-based indices) in favor of the combined BT+PT, this training modality seems to be more suitable for enhancing interlimb postural control.“
(cf. Conclusions): „The greater effectiveness of combined BT+PT in parameters resulting from more sophisticated, in-depth analyses suggest that this training modality is probably better suited to positively influencing interlimb postural control.“**

**Reply: I appreciate the transparent acknowledgment that this was a post-hoc decision. However, I still consider this problematic. Even if motivated by floor- and ceiling-effects, the approach still results in outcome-specific selection of data subsets after inspection of the data. Also, the subsequent prioritization of the CoP-based analyses appears partly post-hoc as well. I still feel that this complicates the interpretation of the findings.**

Response: Thank you for highlighting this aspect again. Consequently, we emphasized the inconsistency across outcome domains more explicitly in the Abstract and Conclusions.

Abstract: *”Due to the floor and ceiling effects across outcome domains, it cannot be concluded that one training mode is superior to the other. However, the more in-depth analysis (i.e., CoP-based indices) yields findings in favor of the combined BT+PT and suggests that regulatory processes, in particular, benefit from this training modality.“*

Conclusions: *”The floor and ceiling effects across outcome domains prevent definitive conclusion regarding the superiority of either training modality. However, the detailed analyses based on CoP-derived indices favor the combined BT+PT approach, suggesting that this training modality is particularly effective in enhancing balance regulatory processes.“*

**COMMENT on L228: The model includes multiple main effects and several interaction terms (including 3-way interactions) with a comparably large sample size for sport science papers, but still relatively small sample size of n = 57). This seems quite complex and raises the concern of the models being overfitted. Are all these interactions theoretically justified? This is especially relevant as some findings are based on bivariate classification of results into significant/non-significant around p-values close to the traditional 0.05-threshold in complex interaction terms. More importantly, many of the key conclusions rely on higher order interactions. For example, effects such as time x group x condition x difficulty level x leg (implicitly decomposed across multiple 3-way terms) are very difficult to interpret. From a physiological perspective, I’m not convinced that such interaction structures are meaningful? This should at least be discussed critically. Moreover, you analyze multiple dependent variables, sometimes after computing the dependent variable as a ratio. I feel that it would strengthen the paper if there would be a clearly defined primary outcome.**

**Response: We agree with the reviewer that the experiment implemented a complex design. The three-factor interactions were expected for observed values (i.e., the basic group x time interaction for any intervention design was expected to be moderated by at least one of the other design factors). Therefore, we opted to specify the expected effects as a priori contrasts (rather than following up significant omnibus F-tests with multiple comparisons; see Hays [16] and Schad et al. [15]. For clarification, we have added the following information to the Material and Methods section (cf. Inferential statistics): “We estimated fixed effects for the two contrasts defined for control and training groups, pretest/posttest, static/dynamic balance condition, task difficulty level, and dominant/non- dominant leg. The first contrast (C1) tested the average of the two training groups against the active CON group; the second contrast (C2) tested the difference between the two training groups. Contrasts with effect coding were used for the other factors. We also included all simple interactions between these main effects and six theoretically relevant three-factor interactions arising from the combination of time × (C1 | C2) × (balance condition | task difficulty level leg). These contrasts and associated interactions were dictated by the design and specified a priori with a clear expectation about the profile of means, not as omnibus F-tests to be followed up with post-hoc t-tests adjusted for multiple comparisons [15,16]. The design was used both for the GLMM for the parameter success rate and the LMM for the CoP-based parameters. For unbalanced designs, (G)LMMs preserve statistical power better than repeated-measures ANOVAs.”**

**Reply: I appreciate the additional clarification regarding the contrast structure and the rationale for the analyses. However, I still remain unconvinced regarding the overall complexity of the statistical models. I understand the argument that the contrasts were specified a priori and dictated by the experimental design. However, this does not fully address the core issue, whether the resulting higher-order interaction structures are physiologically meaningful and interpretable. In particular, many of the central conclusions rely on complex interactions involving combinations of time, intervention, condition, task difficulty, and leg. From a physiological perspective, I still find it difficult to understand why the effects of the intervention should systematically depend on such complex combinations of factors. Moreover, I still feel that the manuscript insufficiently addresses the issue of multiple hypotheses. While the contrasts may have been planned a priori (as written above without a clear physiological explanation for the complex interactions), the manuscript nevertheless tests a large number of interaction effects across multiple dependent variables and partially non-overlapping datasets. This is especially relevant given that several findings are close to the conventional p = 0.05 threshold and the low reliability (see above for the ICC comment), which will lead to interaction estimates becoming unstable. Thus, I still feel that the current analysis substantially increases the likelihood of spurious and non-replicable findings. This is still a major limitation and should be clearly labeled as a limitation.**

Response: We agree with the reviewer that this is a complex experiment with limitations. We have presented a transparent analysis, acknowledged the limitations, and did not want to overstate conclusions. We cannot expect agreement with all our decisions but think that our analyses fall in the space where reasonable people should be allowed to disagree. We will certainly make data and scripts available for alternative analyses.

We comment on two sentences in your comment:

The reviewer writes *“****From a physiological perspective, I still find it difficult to understand why the effects of the intervention should systematically depend on such complex combinations of factors.”*** The dependency is a necessary consequence of any experimental design with pretest/posttest, control and experimental conditions and theoretically motivated additional factors. One alternative is to analyze change scores (i.e., remove the pretest/posttest factor from the interactions). Another conceptually, but not statistically simpler solution is to analyze posttest scores and include pretest as a covariate. These alternatives have their own weaknesses well documented in the literature.

The reviewer also writes: *“****Thus, I still feel the current analysis substantially increases the likelihood of spurious and non-replicable findings. This is still a major limitation and should be clearly labeled as a limitation.”*** We are not in disagreement with this sentiment. In our opinion, the most promising way forward is replication. We think that our experiment and analyses provide a necessary and suitable platform for such a perspective. In accordance with your suggestions, we have added the following major limitation to the respective paragraph (cf. Discussion): *“Ninth, the obtained findings resulted from complex interactions among several independent variables (e.g., group × time × condition/level/leg) based on partially overlapping data, which compromises their meaning and interpretation from a physiological perspective. Furthermore, some findings are close to the conventional p = 0.05 threshold, which increases the likelihood of spurious and non-replicable results.”*

**Responses to Reviewer #3:**

**COMMENT: This is a nicely written manuscript. The rationale is justified, and the outcomes would add to the existing literature. I particularly find the statistical analysis quite sophisticated. However, there are a few concerns and suggestions that I would like to invite the authors to take into account.**

Response: We thank the reviewer for this affirmative comment. Further, we are grateful for the time and effort invested in providing constructive criticism. Below you will find the responses to your comments. All changes made in response to your comments were highlighted in yellow in the revised manuscript.

**COMMENT: Abstract**

**Keywords seem to be missing. The objective is unclear. What do you mean by "of both training modalities"? The training modalities should be explicitly mentioned; otherwise, this leaves the reader unsure about the independent variables, Last statement: This statement reads awkwardly, particularly toward its end.**

Response: Due to journal requirements the keywords are not listed in the manuscript but in the online submission tool and include the following: standing; side-to-side difference; asymmetry; intervention; football; sequential balance assessment.

For clarification the objective was revised and reads as follows (cf. Abstract): *“Thus, we investigated the effects of a single-mode versus combined training modality on interlimb static and dynamic balance performance in soccer players.”*

Further, we have revised the statements as follows (cf. Abstract): *“Both, single-mode BT (binary outcome) and combined BT+PT (metric outcome) appear to be effective in improving interlimb static and dynamic postural control in young male soccer players. Due to the inconsistency across outcome domains, it cannot be concluded that one training mode is superior to the other. However, the more in-depth analysis (i.e., CoP-based indices) yields findings in favor of the combined BT+PT and suggests that regulatory processes, in particular, benefit from this training modality.”*

**COMMENT: Introduction:**

**The rationale is sound. For some reason, part of the content does not exist. Although the study can be classified under incremental science, I find the outcomes, practically speaking, quite interesting.**

Response: We apologize for the formatting error and have corrected it. The entire introduction should now be visible. We would also like to thank you for your comment confirming that the present study is interesting from both a theoretical and a practical perspective.

**COMMENT: Line 63 For some reason, the rest of the text is gone and is not visible.**

Response: As mentioned before, we apologize for the formatting error and have corrected it. The entire introduction should now be visible.

**COMMENT: Methods:**

**Please use body mass instead of weight.**

Response: In accordance with your suggestion, we have changed “weight” to “body mass”.

**COMMENT: Line 264 This is partly inconsistent and redundant with what you mentioned at the beginning of this subsection**

Response: To ensure that the information is consistent and not redundant, the respective statement in line 264 has been deleted.

**COMMENT: Line 295 I would explicitly indicate that the duration of training was matched between the two experimental groups.**

Response: Thank you for highlighting this aspect. In accordance with your suggestion, we have added the following statement to the Material and methods section (cf. Interventions): *“Furthermore, it was ensured that training duration was matched between the groups.”*

**COMMENT: Line 317 No parameters of internal training load were used. This could be added to the limitations.**

Response: We appreciate this important point and agree with the reviewer that no internal training load parameters were measured. This represents a limitation of the present study and has been included in the respective section as follows (cf. Discussion): *“Fifth, no internal training load parameters were measured. Therefore, future studies should be expanded to include the measurement of metabolic (e.g., heart rate, blood lactate) and biomechanical (e.g., joint contact forces, muscle-tendon forces) components.”*

**COMMENT: Line 333 What about those who use both sides equally?**

Response: Thank you for highlighting this aspect. Players who use both sides were asked which side they prefer—that is, which one they use more often. To address the reviewer's comment, the following statement has been added to the Material and methods section (cf. Assessment of balance performance): *„Players who reported using both sides were also asked which side they preferred—that is, which one they used more often.“*

**COMMENT: Line 344 Is this test-retest reliability, with, for instance, a couple of days in between, or between-trial (reps) reliability? Additionally, I don't think an ICC of 0.50 indicates good reliability; at best, it is moderate. Also, the reference (35) used is quite old. You could refer to** [**https://www.sciencedirect.com/science/article/pii/S1556370716000158**](https://www.sciencedirect.com/science/article/pii/S1556370716000158)**. Sorry if I missed it, did participants get familiarised with the tasks?**

Response: Thank you for bringing this to our attention. Between-trial data were used to calculate test-retest reliability. We have added this information to the Material and methods section (cf. Assessment of balance performance). In addition, we have changed “fair to good” to “moderate” reliability and used the suggested reference. Indeed, the players were familiarized with the balance tasks as mentioned in the Material and methods section (cf. Assessment of balance performance): “For each difficulty level, two practice trials were performed to familiarize with the respective test condition followed by […].”

**COMMENT: Line 348 Here, the reliability ranges from poor to moderate according to** [**https://www.sciencedirect.com/science/article/pii/S1556370716000158**](https://www.sciencedirect.com/science/article/pii/S1556370716000158)**. This could be added to the limitations as we can't be confident enough that the effects are due to training and not to poor reliability.**

Response: We agree with the reviewer and have changed “fair to good” to “poor to moderate” reliability and used the suggested reference. In addition, we have added this aspect to the limitations paragraph (cf. Discussion): *“Sixth, between-trial test-retest reliability was poor to moderate for the dynamic balance task. Therefore, it cannot be ruled out that the low repeatability of data collection may have affected the accuracy of the present findings.”*

**COMMENT: Results:**

**The statistical analysis and data interpretation are nicely detailed. The statistical approach used is quite sophisticated.**

Response: We thank the reviewer for this affirmative comment.

**COMMENT: Discussion:**

**I prefer to specify that this is plyometric jump training (PJT). You could use this wording throughout the manuscript instead of the existing.**

Response: We agree with your comment and have specified PT as plyometric jump training in several parts of the manuscript. However, we would like to stick with the abbreviation PT instead of PJT, as this is consistent with previous studies [1-4].

**COMMENT: Line 576 I suggest consistently specifying the sex of participants.**

Response: We agree with your comment and have now consistently specified the gender (i.e., male) of our participants throughout the revised manuscript.

**COMMENT: Line 675 I would also add the poor to moderate reliability of the different parameters as a serious potential source of bias and, therefore, a limitation of this study that may affect the accuracy/veracity of findings. Additionally, the lack of injury monitoring and the potential associated with LSI could be added to the limitations. What about adding the main research perspectives, or follow-up studies that could be conducted?**

Response: In accordance with your suggestions, we have added the poor to moderate reliability of the different parameters as a limitation of this study to the respective paragraph (cf. Discussion): *“Sixth, between-trial test-retest reliability was poor to moderate for the dynamic balance task. Therefore, it cannot be ruled out that the low repeatability of data collection may have affected the accuracy of the present findings.”*

In addition, we have added the lack of injury monitoring and their associations with LSI as a further limitation and added this aspect as a future main research perspective (cf. Discussion): *“Seventh, training-related changes were determined on a performance level but not in terms of injury risk. Consequently, future studies are needed that investigate the relationship between training-related reductions in interlimb asymmetry and injury risk in young soccer players.”*

**References used for revision**

1.	Chaouachi, M.; Granacher, U.; Makhlouf, I.; Hammami, R.; Behm, D.G.; Chaouachi, A. Within session sequence of balance and plyometric exercises does not affect training adaptations with youth soccer athletes. *J Sports Sci Med* **2017**, *16*, 125-136.

2.	Makhlouf, I.; Chaouachi, A.; Chaouachi, M.; Ben Othman, A.; Granacher, U.; Behm, D.G. Combination of agility and plyometric training provides similar training benefits as combined balance and plyometric training in young soccer players. *Front Physiol* **2018**, *9*, 1611, doi:10.3389/fphys.2018.01611.

3.	Muehlbauer, T.; Borgmann, K.; Limpach, S.; Krombholz, D.; Panzer, S. Impact of unilateral single-mode balance training vs. combined balance and plyometric training on soccer players' interlimb asymmetry in balance and neuromuscular performance. *Front Sports Act Living* **2026**, *8*, 1743152, doi:10.3389/fspor.2026.1743152.

4.	Muehlbauer, T.; Wagner, V.; Brueckner, D.; Schedler, S.; Schwiertz, G.; Kiss, R.; Hagen, M. Effects of a blocked versus an alternated sequence of balance and plyometric training on physical performance in youth soccer players. *BMC Sports Sci Med Rehabil* **2019**, *11*, 18, doi:10.1186/s13102-019-0131-y.
