# Supplementary material for: Effects of unilateral single-mode balance training compared to combined balance and plyometric training on soccer players’ interlimb asymmetry in static and dynamic balance performance
Source: PLoS One. 2026 Sep 16;21(9):e0358565. doi: 10.1371/journal.pone.0358565 (PMC13581024; doi:10.1371/journal.pone.0358565)
Supplement: Peer review history

# Peer review: Decision Letter 0

**DOI:** 10.1371/journal.pone.0358565.r001

**Authors:** Eduard Kurz

26 Mar 2026

Dear Dr. Muehlbauer,

Thank you for submitting your manuscript to PLOS ONE. After careful consideration, we feel that it has merit but does not fully meet PLOS ONE’s publication criteria as it currently stands. Therefore, we invite you to submit a revised version of the manuscript that addresses the points raised during the review process.

Please submit your revised manuscript by May 10 2026 11:59PM. If you will need more time than this to complete your revisions, please reply to this message or contact the journal office at plosone@plos.org. When you're ready to submit your revision, log on to https://www.editorialmanager.com/pone/ and select the 'Submissions Needing Revision' folder to locate your manuscript file.

- A letter that responds to each point raised by the academic editor and reviewer(s). You should upload this letter as a separate file labeled 'Response to Reviewers'.
- A marked-up copy of your manuscript that highlights changes made to the original version. You should upload this as a separate file labeled 'Revised Manuscript with Track Changes'.
- An unmarked version of your revised paper without tracked changes. You should upload this as a separate file labeled 'Manuscript'.

If you would like to make changes to your financial disclosure, please include your updated statement in your cover letter. Guidelines for resubmitting your figure files are available below the reviewer comments at the end of this letter.

If applicable, we recommend that you deposit your laboratory protocols in protocols.io to enhance the reproducibility of your results. Protocols.io assigns your protocol its own identifier (DOI) so that it can be cited independently in the future. For instructions see: https://journals.plos.org/plosone/s/submission-guidelines#loc-laboratory-protocols. Additionally, PLOS ONE offers an option for publishing peer-reviewed Lab Protocol articles, which describe protocols hosted on protocols.io. Read more information on sharing protocols at https://plos.org/protocols?utm_medium=editorial-email&utm_source=authorletters&utm_campaign=protocols.

As the corresponding author, your ORCID iD is verified in the submission system and will appear in the published article. PLOS supports the use of ORCID, and we encourage all coauthors to register for an ORCID iD and use it as well. Please encourage your coauthors to verify their ORCID iD within the submission system before final acceptance, as unverified ORCID iDs will not appear in the published article. *Only*  the individual author can complete the verification step; PLOS staff *cannot*  verify ORCID iDs on behalf of authors.

We look forward to receiving your revised manuscript.

Kind regards,

Eduard Kurz, PhD

Academic Editor

PLOS One

**Journal Requirements:**

When submitting your revision, we need you to address these additional requirements.

1. Please ensure that your manuscript meets PLOS ONE's style requirements, including those for file naming. The PLOS ONE style templates can be found at

https://journals.plos.org/plosone/s/file?id=wjVg/PLOSOne_formatting_sample_main_body.pdf and

https://journals.plos.org/plosone/s/file?id=ba62/PLOSOne_formatting_sample_title_authors_affiliations.pdf

2. Thank you for stating the following financial disclosure:

“This study was funded by a grant from the Deutsche Forschungsgemeinschaft (DFG; German Research Foundation) – grant numbers: MU 3327/5-1 and PA 774/21-1. The support by the Open Access Publication Fund of the University of Duisburg-Essen is acknowledged. The funding body is independent of the design of the study and collection, analysis, and interpretation of data and in writing the manuscript. Open access funding enabled and organized by the project DEAL.”

Please state what role the funders took in the study.  If the funders had no role, please state: "The funders had no role in study design, data collection and analysis, decision to publish, or preparation of the manuscript."

If this statement is not correct you must amend it as needed.

Please include this amended Role of Funder statement in your cover letter; we will change the online submission form on your behalf.

3. Please note that funding information should not appear in any section or other areas of your manuscript. We will only publish funding information present in the Funding Statement section of the online submission form. Please remove any funding-related text from the manuscript.

4. Your ethics statement should only appear in the Methods section of your manuscript. If your ethics statement is written in any section besides the Methods, please delete it from any other section.

5. Please upload a new copy of Figures 1, 3, 4, and 5 as the detail is not clear. Please follow the link for more information:  https://journals.plos.org/plosone/s/figures

6. We note that Figure 2 includes an image of a participant in the study.

As per the PLOS ONE policy (http://journals.plos.org/plosone/s/submission-guidelines#loc-human-subjects-research) on papers that include identifying, or potentially identifying, information, the individual(s) or parent(s)/guardian(s) must be informed of the terms of the PLOS open-access (CC-BY) license and provide specific permission for publication of these details under the terms of this license. Please download the Consent Form for Publication in a PLOS Journal (http://journals.plos.org/plosone/s/file?id=8ce6/plos-consent-form-english.pdf). The signed consent form should not be submitted with the manuscript, but should be securely filed in the individual's case notes. Please amend the methods section and ethics statement of the manuscript to explicitly state that the patient/participant has provided consent for publication: “The individual in this manuscript has given written informed consent (as outlined in PLOS consent form) to publish these case details”.

If you are unable to obtain consent from the subject of the photograph, you will need to remove the figure and any other textual identifying information or case descriptions for this individual.

7. We note that Figure 2 in your submission contain copyrighted images. All PLOS content is published under the Creative Commons Attribution License (CC BY 4.0), which means that the manuscript, images, and Supporting Information files will be freely available online, and any third party is permitted to access, download, copy, distribute, and use these materials in any way, even commercially, with proper attribution. For more information, see our copyright guidelines: http://journals.plos.org/plosone/s/licenses-and-copyright.

We require you to either (1) present written permission from the copyright holder to publish these figures specifically under the CC BY 4.0 license, or (2) remove the figures from your submission:

1. You may seek permission from the original copyright holder of Figure 2 to publish the content specifically under the CC BY 4.0 license.

We recommend that you contact the original copyright holder with the Content Permission Form (http://journals.plos.org/plosone/s/file?id=7c09/content-permission-form.pdf) and the following text:

“I request permission for the open-access journal PLOS ONE to publish XXX under the Creative Commons Attribution License (CCAL) CC BY 4.0 (http://creativecommons.org/licenses/by/4.0/). Please be aware that this license allows unrestricted use and distribution, even commercially, by third parties. Please reply and provide explicit written permission to publish XXX under a CC BY license and complete the attached form.”

Please upload the completed Content Permission Form or other proof of granted permissions as an "Other" file with your submission.

In the figure caption of the copyrighted figure, please include the following text: “Reprinted from [ref] under a CC BY license, with permission from [name of publisher], original copyright [original copyright year].”

2. If you are unable to obtain permission from the original copyright holder to publish these figures under the CC BY 4.0 license or if the copyright holder’s requirements are incompatible with the CC BY 4.0 license, please either i) remove the figure or ii) supply a replacement figure that complies with the CC BY 4.0 license. Please check copyright information on all replacement figures and update the figure caption with source information. If applicable, please specify in the figure caption text when a figure is similar but not identical to the original image and is therefore for illustrative purposes only.

8. If the reviewer comments include a recommendation to cite specific previously published works, please review and evaluate these publications to determine whether they are relevant and should be cited. There is no requirement to cite these works unless the editor has indicated otherwise.

[Note: HTML markup is below. Please do not edit.]

Reviewers' comments:

Reviewer's Responses to Questions

**Comments to the Author**

1. Is the manuscript technically sound, and do the data support the conclusions?

Reviewer #1: Partly

Reviewer #2: Partly

**********

2. Has the statistical analysis been performed appropriately and rigorously?

Reviewer #1: No

Reviewer #2: Yes

**********

3. Have the authors made all data underlying the findings in their manuscript fully available?

The PLOS Data policy

Reviewer #1: No

Reviewer #2: Yes

**********

4. Is the manuscript presented in an intelligible fashion and written in standard English?

Reviewer #1: Yes

Reviewer #2: Yes

**********

Reviewer #1: Overall Evaluation

This manuscript investigates the effects of unilateral training modalities on interlimb asymmetry in static and dynamic postural control in elite youth soccer players. The study is conducted within a high-level academy setting, the intervention is well structured, and implementation during the competitive season represents a clear applied strength.

The topic is relevant, particularly in the context of neuromuscular performance and asymmetry modulation in youth sport. The dataset appears carefully collected and the study is generally well organized.

However, several conceptual, methodological, and transparency-related issues require clarification and strengthening before the manuscript can be considered for publication.

For these reasons, I recommend major revision.

The required revisions primarily concern:

• transparency regarding overlap with previously published data,

• conceptual justification of asymmetry reduction,

• statistical handling of cluster randomization and maturation,

• cautious interpretation of practical relevance,

• and a more conservative framing of the discussion and conclusions.

Major Comments

Transparency regarding previously published data

A closely related study by the same authors using the identical intervention cohort (elite youth soccer players from SG Dynamo Dresden; 9-week unilateral BT vs. BT+PT vs. control; YBT-LQ, SLDL, EMG; LSI-based analyses) has recently been published.

Given the apparent overlap in:

• population,

• intervention design,

• measurement tools,

• and asymmetry-based outcome analyses,

it is essential that the authors explicitly clarify:

1. Whether the present manuscript uses the same dataset.

2. If so, which analyses are novel and how they substantively differ from the previously published work.

3. That the prior publication is transparently cited and clearly distinguished.

This is not inherently problematic provided that the research questions and analytical focus are clearly distinct. However, explicit clarification is required to avoid concerns regarding redundant publication or insufficient differentiation between manuscripts.

Conceptual justification of interlimb asymmetry reduction

The manuscript implicitly assumes that reducing interlimb asymmetry is beneficial. This assumption requires stronger theoretical grounding.

Key issues that should be addressed:

• Are asymmetries inherently maladaptive in elite youth soccer?

• Could certain asymmetries be functionally adaptive due to sport-specific demands (e.g., dominant kicking leg vs. stance leg)?

• Is there direct evidence that reducing asymmetry improves performance or reduces injury risk specifically in elite youth soccer players?

• What is the baseline asymmetry magnitude in this cohort, and is it clinically meaningful?

Currently, the injury-risk argument relies largely on basketball or mixed-athlete populations. Transferability to elite youth soccer requires more careful discussion.

A more differentiated perspective on adaptive vs. maladaptive asymmetries would substantially strengthen the theoretical framework.

Practical and clinical relevance

Although statistically significant reductions in LSI were observed, the practical relevance remains unclear.

Please clarify:

• Did the changes move players below established risk thresholds?

• Are the observed reductions large enough to meaningfully influence injury risk?

• Were injury data analyzed in relation to asymmetry changes?

At present, the conclusions may overstate practical implications. A clearer distinction between statistical significance and applied relevance is necessary.

Cluster randomization and statistical modelling

The manuscript states that cluster randomization was applied.

However, the statistical analyses appear to rely on repeated measures ANOVA without modelling potential cluster effects.

Please clarify:

• What defined a cluster (team, age group, subgroup)?

• Was clustering accounted for statistically?

• If not, provide justification or consider reanalysis using appropriate mixed-effects modelling.

Failure to account for clustering may affect inference validity.

Biological maturation

The inclusion of U13–U17 players introduces substantial variability in biological maturation.

Although maturity offset is reported, it does not appear to be included as a covariate in the analyses.

Please clarify:

• Was maturation statistically controlled?

• Could observed changes reflect developmental processes rather than intervention effects?

Given the age range, maturation is a relevant potential confounder.

Task specificity and transfer

Balance training adaptations are often task-specific with limited transfer between tasks.

The manuscript would benefit from elaboration on:

• Why improvements in YBT-LQ and SLDL are expected to generalize to soccer performance or injury prevention.

• The ecological validity of these assessments in elite youth soccer.

Addressing specificity versus transfer would strengthen the interpretation.

Discussion and Conclusion: Conceptual Contribution and Tone

While the study is well conducted and addresses a relevant applied question, its conceptual contribution appears primarily incremental rather than novel.

The findings confirm that unilateral balance-based training can modulate interlimb asymmetry — an effect that has been previously reported in other populations and settings. The present study adds valuable applied evidence from an elite youth soccer context and compares two unilateral training modalities within the same cohort. However, it does not establish a fundamentally new theoretical framework regarding asymmetry, neuromuscular adaptation, or injury prevention.

For this reason, I recommend that the Discussion and Conclusion be written in a more conservative tone.

Specifically:

• Avoid overstating novelty.

• Avoid strong injury-prevention implications, as injury reduction was not directly demonstrated.

• Position the contribution as applied extension rather than conceptual advancement.

• Clearly distinguish between statistical findings and broader performance or injury claims.

Reframing the conclusions as incremental applied evidence would improve scientific balance and credibility.

Title

The current title (“Role of training”) is rather broad and does not adequately reflect:

• the unilateral intervention design,

• the comparative training modalities,

• and the static vs. dynamic postural control focus.

A more precise title would better represent the study and clearly differentiate it from related previously published work.

Specific Comments

Abstract

• Strengthen the applied relevance (injury prevention and/or performance context).

• Clarify why comparing single-mode and combined training modalities is important.

• Specify playing level (elite academy).

• Indicate whether the intervention was conducted in-season.

• Elaborate briefly on the practical meaning of the observed improvements.

Introduction

• Provide sport-specific evidence for youth soccer rather than relying predominantly on basketball data.

• Clarify whether clinically meaningful asymmetries are documented in elite youth soccer.

• Discuss adaptive vs. maladaptive asymmetry.

• Address whether balance adaptations persist under fatigue conditions.

Methods

• Clarify details of cluster randomization.

• Clarify whether sitting height was measured.

• Specify how maturation was balanced and handled statistically.

Intervention

• Clarify whether plyometric exercises were performed unilaterally or bilaterally.

• Specify take-off and landing leg.

• Ensure key unilateral aspects are clearly stated in the abstract.

• Provide sufficient detail for reproducibility.

Minor Points

• Consider adjustment for multiple comparisons.

• Ensure consistent terminology regarding asymmetry thresholds.

Final Recommendation

The manuscript addresses an interesting and applied question within elite youth soccer and is based on a valuable dataset collected in a real-world setting.

However, conceptual clarification, methodological transparency, and more conservative interpretation are required before publication.

For these reasons, I recommend major revision.

Reviewer #2: Dear editor, dear authors,

Thank you for the opportunity to review this manuscript. The study addresses a relevant topic with a considerable sample size, and the randomized design is appreciated. However, I have several concerns regarding the conceptual framing and the statistical approach. Please see specific comments below:

Abstract

The conclusion reads that the combined BT+PT is “presumably more suitable” . However, the results show that BT is superior for success rate (although they performed significantly worse during pre), BT+PT is superior for CoP, and that there are no differences for LSI. Thus, this does not seem fully consistent with the conclusion. Therefore, I would recommend toning this down and presenting a more balanced interpretation.

Methods/Results

L102: Participants were allocated to the training groups using cluster randomization across teams. I wonder, why you did not account for or adjust for the clustering approach in your statistical model, as this might violate independence assumptions and thus inflate your results?

L144: The control group performed stretching exercises including the lower body. However, stretching may also influence proprioception and balance; especially when performed at a single-legged stance. Thus, the control condition might have been not entirely neutral.

L152: Participants were exhibited to a high amount of regular training (soccer + athletic + PE), which might also have included elements comparable to the additional intervention. Thus, it might be hard to isolate the contribution of the relatively low amount of additional training of the intervention (2x 30 min/w)?

L176: Six seconds seems to be rather short? Could you provide reliability data for this test?

L181: The adaptive difficulty approach is interesting, but as difficulty is increased individually in relation to the participants performance level, participants are effectively tested under different conditions, which may reduce comparability between subjects. Could you clarify how this affects interpretability of group differences?

L190: Was there a familiarization session prior to data collection? Moreover, you state that a trial was discarded and repeated if participants failed. What was the min and max number of trials per participants? Moreover, later in L210/211 you mention that the maximum number of valid trials was 3 and that the LSI was computed as min/max. Does that mean that for some participants you used a lower number than 3 trials to determine min and max?

L195: Is r = 0.57 really an indicator for sufficient validity (~ 32% of variance explained)? Also, the criterion (against what was the UST tested?) should be specified. Moreover, the ICC should be reported and interpreted by including its 95%CI.

L252: While I understand the argument for restricting the analysis for the success rate to levels 5-6 and the CoP to levels 3-4, this seems to be a post-hoc decision based on the distribution of your data, rather than an apriori decision. Moreover, this results in different datasets for different outcomes and makes it more difficult to compare between the different outcomes.

L228: The model includes multiple main effects and several interaction terms (including 3-way interactions) with a comparably large sample size for sport science papers, but still relatively small sample size of n = 57). This seems quite complex and raises the concern of the models being overfitted. Are all these interactions theoretically justified? This is especially relevant as some findings are based on bivariate classification of results into significant/non-significant around p-values close to the traditional 0.05-threshold in complex interaction terms. More importantly, many of the key conclusions rely on higher-order interactions. For example, effects such as time x group x condition x difficulty level x leg (implicitly decomposed across multiple 3-way terms) are very difficult to interpret. From a physiological perspective, I’m not convinced that such interaction structures are meaningful? This should at least be discussed critically. Moreover, you analyze multiple dependent variables, sometimes after computing the dv as a ratio. I feel that it would strengthen the paper if there would be a clearly defined primary outcome.

Table 2: There is a typo (BP+PT instead of BT+PT)

**********

what does this mean?). If published, this will include your full peer review and any attached files.

If you choose “no”, your identity will remain anonymous but your review may still be made public.

**Do you want your identity to be public for this peer review?**  For information about this choice, including consent withdrawal, please see our Privacy Policy

Reviewer #1: **Yes:** Ralf Roth

Reviewer #2: No

**********

[NOTE: If reviewer comments were submitted as an attachment file, they will be attached to this email and accessible via the submission site. Please log into your account, locate the manuscript record, and check for the action link "View Attachments". If this link does not appear, there are no attachment files.]

To ensure your figures meet our technical requirements, please review our figure guidelines: https://journals.plos.org/plosone/s/figures

You may also use PLOS’s free figure tool, NAAS, to help you prepare publication quality figures: https://journals.plos.org/plosone/s/figures#loc-tools-for-figure-preparation.

NAAS will assess whether your figures meet our technical requirements by comparing each figure against our figure specifications.

# Author comment: Author response to Decision Letter 1

**DOI:** 10.1371/journal.pone.0358565.r002

6 May 2026

Manuscript Number: PONE-D-26-01855

Title: Interlimb static and dynamic postural control in young soccer players: Role of training

Responses to Journal Requirements:

Comment 1: Please ensure that your manuscript meets PLOS ONE's style requirements, including those for file naming.

Response: We have checked the PLOS ONE's style requirements and confirm that they are met including those for file naming.

Comment 2: 2. Thank you for stating the following financial disclosure:

“This study was funded by a grant from the Deutsche Forschungsgemeinschaft (DFG; German Research Foundation) – grant numbers: MU 3327/5-1 and PA 774/21-1. The support by the Open Access Publication Fund of the University of Duisburg-Essen is acknowledged. The funding body is independent of the design of the study and collection, analysis, and interpretation of data and in writing the manuscript. Open access funding enabled and organized by the project DEAL.” Please state what role the funders took in the study. If the funders had no role, please state: "The funders had no role in study design, data collection and analysis, decision to publish, or preparation of the manuscript." If this statement is not correct you must amend it as needed. Please include this amended Role of Funder statement in your cover letter; we will change the online submission form on your behalf.

Response: We hereby confirm that the funders had no role and have confirmed this in the cover letter.

Comment 3: Please note that funding information should not appear in any section or other areas of your manuscript. We will only publish funding information present in the Funding Statement section of the online submission form. Please remove any funding-related text from the manuscript.

Response: In accordance with your suggestion, we have removed any funding-related text from the manuscript.

Comment 4: Your ethics statement should only appear in the Methods section of your manuscript. If your ethics statement is written in any section besides the Methods, please delete it from any other section.

Response: In accordance with your suggestion, we now only provide the ethics statement in the Material and Method section (cf. Participants) as follows: “Ethical permission (approval number: TM_04.06.2020) was given by the Human Ethics Committee at the University of Duisburg-Essen, Faculty of Educational Sciences. Participants’ assent and parents’ written informed consent were obtained prior to the start of the study.” This change and all other changes in response to the comments related to Journal Requirements are highlighted in purple.

Comment 5: Please upload a new copy of Figures 1, 3, 4, and 5 as the detail is not clear.

Response: In accordance with your suggestion, we have uploaded a new copy of Figures 1, 3, 4, and 5.

Comment 6: We note that Figure 2 includes an image of a participant in the study.

Response: The participant shown in Figure 2 does not represent an image of a participant in the study but was created by using ChatGPT (OpenAI, San Francisco, CA). This information has been added to the Material and Method section (cf. Participants) as follows: “The individual shown in this diagram was created by using ChatGPT (OpenAI, San Francisco, CA).” and is now mentioned in the Generative AI statement: “Further, ChatGPT (OpenAI, San Francisco, CA) was used to create Figure 2.”

Comment 7: We note that Figure 2 in your submission contains copyrighted images. All PLOS content is published under the Creative Commons Attribution License (CC BY 4.0), which means that the manuscript, images, and Supporting Information files will be freely available online, and any third party is permitted to access, download, copy, distribute, and use these materials in any way, even commercially, with proper attribution.

Response: As stated in response to the previous comment, Figure 2 was created by using ChatGPT (OpenAI, San Francisco, CA) and does not contain copyrighted images.

Comment 8: If the reviewer comments include a recommendation to cite specific previously published works, please review and evaluate these publications to determine whether they are relevant and should be cited. There is no requirement to cite these works unless the editor has indicated otherwise.

Response: The reviewers’ comments do not include a recommendation to cite specific previously published works.

Responses to Reviewer #1:

Comment: This manuscript investigates the effects of unilateral training modalities on interlimb asymmetry in static and dynamic postural control in elite youth soccer players. The study is conducted within a high-level academy setting, the intervention is well structured, and implementation during the competitive season represents a clear applied strength. The topic is relevant, particularly in the context of neuromuscular performance and asymmetry modulation in youth sport. The dataset appears carefully collected and the study is generally well organized.

However, several conceptual, methodological, and transparency-related issues require clarification and strengthening before the manuscript can be considered for publication.

For these reasons, I recommend major revision.

The required revisions primarily concern:

• transparency regarding overlap with previously published data,

• conceptual justification of asymmetry reduction,

• statistical handling of cluster randomization and maturation,

• cautious interpretation of practical relevance,

• and a more conservative framing of the discussion and conclusions.

Response: We thank the reviewer for summarizing the manuscript and for this affirmative comment. Further, we are grateful for the time and effort invested in providing constructive criticism. Below you will find the responses to your comments. All changes made in response to your comments were highlighted in yellow in the revised manuscript.

Major Comments

Transparency regarding previously published data.

A closely related study by the same authors using the identical intervention cohort (elite youth soccer players from SG Dynamo Dresden; 9-week unilateral BT vs. BT+PT vs. control; YBT-LQ, SLDL, EMG; LSI-based analyses) has recently been published.

Given the apparent overlap in:

• population,

• intervention design,

• measurement tools,

• and asymmetry-based outcome analyses,

it is essential that the authors explicitly clarify:

1. Whether the present manuscript uses the same dataset.

2. If so, which analyses are novel and how they substantively differ from the previously published work.

3. That the prior publication is transparently cited and clearly distinguished.

This is not inherently problematic provided that the research questions and analytical focus are clearly distinct. However, explicit clarification is required to avoid concerns regarding redundant publication or insufficient differentiation between manuscripts.

Response: We apologize for being imprecise. The dataset in this manuscript with N=57 participants is similar but not identical to that from a previous study with N=64 participants [1], as it includes only some of the same participants. This is because not all participants from the previous study were able to complete the progressively increasing task difficulty in the present study. In other words, the population is similar, the intervention design is identical, but the measurement tools (present study: unipedal stance with/without leg swing vs. previous study: Y Balance Test – Lower Quarter, Single leg drop landing test) and the asymmetry-based outcome analyses (present study: success rate, posttest – pretest of log, ap-/ml-LSI vs. previous study: YBT-LQ-/SLDL-LSI) are different. For clarification, we have added the following information to the Material and Methods section (cf. Data analyses): “Some of the data originate from a previously published study [1] in which the same interventions were used but different assessments were conducted.”

Comment: Conceptual justification of interlimb asymmetry reduction.

The manuscript implicitly assumes that reducing interlimb asymmetry is beneficial. This assumption requires stronger theoretical grounding.

Key issues that should be addressed:

• Are asymmetries inherently maladaptive in elite youth soccer?

• Could certain asymmetries be functionally adaptive due to sport-specific demands (e.g., dominant kicking leg vs. stance leg)?

• Is there direct evidence that reducing asymmetry improves performance or reduces injury risk specifically in elite youth soccer players?

• What is the baseline asymmetry magnitude in this cohort, and is it clinically meaningful?

Currently, the injury-risk argument relies largely on basketball or mixed-athlete populations. Transferability to elite youth soccer requires more careful discussion.

A more differentiated perspective on adaptive vs. maladaptive asymmetries would substantially strengthen the theoretical framework.

Response: The studies by Grindem et al. [2] and Logerstedt et al. [3] indicate that interlimb asymmetry values >10% are clinically meaningful, and it seems reasonable to consider this threshold as an indication that deviates from the normal pattern and is therefore maladaptive.

Further, we agree with the reviewer that interlimb asymmetry in postural control arises, among others, from the long-standing preferential use of one limb to perform balance requirements [4], and due to the fact that passing and kicking in soccer are preferably performed with the same leg while keeping balance with the other leg, it is referred to as “functional asymmetry” [5]. However, Gonzalez-Fernandez et al. [6] reported that even amateur soccer players achieve high levels of interlimb asymmetry (i.e., 5.3–12.0%). Accordingly, interventions aiming to reduce interlimb asymmetry should be conducted at an early stage (in youth athletes).

There is evidence that interventions have the potential to reduce interlimb asymmetry [7] and improve neuromuscular parameters [8]. However, no such study exists for soccer. Therefore, as part of a project funded by the German Research Foundation (grant numbers: MU 3327/5-1 and PA 774/21-1), we have investigated the role of single-mode versus combined training on youth soccer players’ interlimb asymmetry in balance and neuromuscular performance. The results showed significant improvements in interlimb asymmetry and muscle activity [1]. The present manuscript continues this line of research and analyses, using a three-step approach (first step: binary parameters; second step: CoP-based indices; third step: LSI indices), the effects of single-mode versus combined training on youth soccer players’ interlimb asymmetry in static and dynamic balance performance. However, future studies are needed to confirm or reject our findings and to address additional aspects (e.g., fatigue). In the latter case, a proposal for a follow-up study has been submitted to the German Research Foundation and is currently under review.

To date, there is no evidence for a link between reduced interlimb asymmetry and injury risk in elite youth soccer players. This is likely because such findings would require epidemiological studies with relatively large sample sizes conducted over a long period of time. We have addressed this point in the Limitations section as follows: “Fifth, training-related changes were determined on a performance level but not in terms of injury risk. Consequently, future studies are needed that investigate the relationship between training-related reductions in interlimb asymmetry and injury risk in young soccer players.”

In terms of baseline asymmetry magnitude in this cohort, we have revised our previous statement in the Discussion section as follows “Regardless of the training modality, a statistically significant increase in LSI values from ≈85% (pretest) to ≈90% (posttest) was observed (see Fig 5 C), indicating a clinically meaningful shift from interlimb asymmetry (>10%) to interlimb asymmetry (≤10%) in postural control.”

With regard to the injury-risk argument, we have now reported findings from a study [9] that investigated different sports including soccer.

Comment: Practical and clinical relevance

Although statistically significant reductions in LSI were observed, the practical relevance remains unclear.

Please clarify:

• Did the changes move players below established risk thresholds?

• Are the observed reductions large enough to meaningfully influence injury risk?

• Were injury data analyzed in relation to asymmetry changes?

At present, the conclusions may overstate practical implications. A clearer distinction between statistical significance and applied relevance is necessary.

Response: Thank you for the valuable feedback. In terms of risk threshold, we have revised our statement in the Discussion section as follows “Regardless of the training modality, a statistically significant increase in LSI values from ≈85% (pretest) to ≈90% (posttest) was observed (see Fig 5 C), indicating a clinically meaningful shift from interlimb asymmetry (>10%) to interlimb asymmetry (≤10%) in postural control.”

Concerning your question, whether the observed reductions are large enough to be rated as meaningfully with respect to injury risk, we refer to the findings reported by Grindem et al. [2] and Logerstedt et al. [3] as their results suggest that interlimb asymmetries exceeding 10% are clinically meaningful. Accordingly, it appears justified to interpret this threshold as a deviation from typical movement patterns and thus may influence injury risk.

However, we did not analyze injury data in relation to changes in interlimb asymmetry, which is an important aim for future studies. Consequently, we have addressed this point in the Limitations section as follows: “Fifth, training-related changes were determined on a performance level but not in terms of injury risk. Consequently, future studies are needed that investigate the relationship between training-related reductions in interlimb asymmetry and injury risk in young soccer players.”

Further, we have revised the conclusions both in the Abstract and the Manuscript as follows.

Cf. Abstract: “[…] Given the greater improvements in parameters obtained from the more in-depth analyses (i.e., CoP-based indices) in favor of the combined BT+PT, this training modality seems to be more suitable for enhancing interlimb postural control. From a practical perspective, the improvements in CoP-based indices suggest that processes of balance regulation in particular benefit from the combined BT+PT.”

Cf. Manuscript: “[…] The greater effectiveness of combined BT+PT in parameters resulting from more sophisticated, in-depth analyses suggest that this training modality is probably better suited to positively influencing interlimb postural control.”

Comment: Cluster randomization and statistical modelling

The manuscript states that cluster randomization was applied.

However, the statistical analyses appear to rely on repeated measures ANOVA without modelling potential cluster effects.

Please clarify:

• What defined a cluster (team, age group, subgroup)?

• Was clustering accounted for statistically?

• If not, provide justification or consider reanalysis using appropriate mixed-effects modelling.

Failure to account for clustering may affect inference validity.

Response: We apologize for lack of precision. Players came from three age groups (squads) from one team (i.e., SG Dynamo Dresden e.V.). With “cluster randomization” we referred to the random assignment of groups of 6–8 players per age group (squad) to one of the three interventions. This approach was used to ensure an even distribution of players across age categories and thus to rule out the influence of chronological age. Thus, neither team nor age group nor randomization cluster could be included as a random factor in the (G)LMMs; they would have only 1, 3, and levels, respectively. For clarification, we added the following information to the Material and Methods section (cf. Participants): “To guard against potential confounding effects of between-subject differences in chronological age, maturity offset, and intervention type, we randomly assigned groups of 6–8 players per age group (i.e., U13, U15, U17 squ

Attachment Submitted filename: response to reviewers.docx

# Peer review: Decision Letter 1

**DOI:** 10.1371/journal.pone.0358565.r003

**Authors:** Eduard Kurz

29 Jun 2026

Dear Dr. Muehlbauer,

Thank you for submitting your manuscript to PLOS ONE. After careful consideration, we feel that it has merit but does not fully meet PLOS ONE’s publication criteria as it currently stands. Therefore, we invite you to submit a revised version of the manuscript that addresses the points raised during the review process.

Please submit your revised manuscript by Aug 13 2026 11:59PM. If you will need more time than this to complete your revisions, please reply to this message or contact the journal office at plosone@plos.org. When you're ready to submit your revision, log on to https://www.editorialmanager.com/pone/ and select the 'Submissions Needing Revision' folder to locate your manuscript file.

- A letter that responds to each point raised by the academic editor and reviewer(s). You should upload this letter as a separate file labeled 'Response to Reviewers'.
- A marked-up copy of your manuscript that highlights changes made to the original version. You should upload this as a separate file labeled 'Revised Manuscript with Track Changes'.
- An unmarked version of your revised paper without tracked changes. You should upload this as a separate file labeled 'Manuscript'.

If you would like to make changes to your financial disclosure, please include your updated statement in your cover letter. Guidelines for resubmitting your figure files are available below the reviewer comments at the end of this letter.

If applicable, we recommend that you deposit your laboratory protocols in protocols.io to enhance the reproducibility of your results. Protocols.io assigns your protocol its own identifier (DOI) so that it can be cited independently in the future. For instructions see: https://journals.plos.org/plosone/s/submission-guidelines#loc-laboratory-protocols. Additionally, PLOS ONE offers an option for publishing peer-reviewed Lab Protocol articles, which describe protocols hosted on protocols.io. Read more information on sharing protocols at https://plos.org/protocols?utm_medium=editorial-email&utm_source=authorletters&utm_campaign=protocols.

As the corresponding author, your ORCID iD is verified in the submission system and will appear in the published article. PLOS supports the use of ORCID, and we encourage all coauthors to register for an ORCID iD and use it as well. Please encourage your coauthors to verify their ORCID iD within the submission system before final acceptance, as unverified ORCID iDs will not appear in the published article. *Only*  the individual author can complete the verification step; PLOS staff *cannot*  verify ORCID iDs on behalf of authors.

We look forward to receiving your revised manuscript.

Kind regards,

Eduard Kurz, PhD

Academic Editor

PLOS One

Journal Requirements:

If the reviewer comments include a recommendation to cite specific previously published works, please review and evaluate these publications to determine whether they are relevant and should be cited. There is no requirement to cite these works unless the editor has indicated otherwise.

Additional Editor Comments:

Dear Prof. Muehlbauer,

Thank you for resubmitting your manuscript. The re-evaluation took a little longer than expected because one of the original reviewers declined to review the manuscript again. It was not easy to find a reviewer who agreed to assess the article in the second round. Finally, we succeeded. Please find the assessments from both reviewers attached. You will notice that the reviewers hold differing opinions regarding the statistics; however, I tend to agree with the second reviewer. Therefore, I ask you to address this point in the limitations section.

I look forward to receiving your revised manuscript.

Sincerely,

Eduard Kurz

[Note: HTML markup is below. Please do not edit.]

Reviewers' comments:

Reviewer's Responses to Questions

**Comments to the Author**

Reviewer #2: (No Response)

Reviewer #3: (No Response)

**********

2. Is the manuscript technically sound, and do the data support the conclusions?

Reviewer #2: Partly

Reviewer #3: (No Response)

**********

3. Has the statistical analysis been performed appropriately and rigorously?

Reviewer #2: N/A

Reviewer #3: (No Response)

**********

4. Have the authors made all data underlying the findings in their manuscript fully available?

The PLOS Data policy

Reviewer #2: Yes

Reviewer #3: (No Response)

**********

5. Is the manuscript presented in an intelligible fashion and written in standard English?

Reviewer #2: Yes

Reviewer #3: (No Response)

**********

Reviewer #2: Dear editorial board, dear authors,

I highly appreciate your revised manuscript! Although the manuscript improved, I have some further comments regarding the responses to my initial points. Please see specific comments below:

Comment: Abstract

The conclusion reads that the combined BT+PT is “presumably more suitable”. However, the results show that BT is superior for success rate (although they performed significantly worse during pre), BT+PT is superior for CoP, and that there are no differences for LSI. Thus, this does not seem fully consistent with the conclusion. Therefore, I would recommend toning this down and presenting a more balanced interpretation.

Response: We agree with the reviewer. We think the results of the in-depth analyses are particularly relevant. These are CoP-based indices that provide insight into the regulatory processes underlying balance performance. We have therefore changed the statement as follows: ”Given the greater improvements in parameters obtained from the more in-depth analyses (i.e., CoP-based indices) in favor of the combined BT+PT, this training modality seems to be more suitable for enhancing interlimb postural control.“

Reply: Even though this is now clearer and toned down in wording, I still feel that the manuscript selectively prioritizes the CoP-based outcomes over the other outcome domains. While I agree that CoP-based measures may provide a more detailed assessment of postural regulation, this does not necessarily make them more meaningful or more valid than the other outcomes. Importantly, superiority of BT+PT is still mainly concluded based on the outcome domain favoring this intervention, whereas the binary outcome favored BT and LSI outcomes showed largely no differences. Thus, I would still recommend presenting the findings in a more balanced manner and emphasizing the inconsistency across outcome domains more explicitly.

Comment: L152: Participants were exhibited to a high amount of regular training (soccer + athletic + PE), which might also have included elements comparable to the additional intervention. Thus, it might be hard to isolate the contribution of the relatively low amount of additional training of the intervention (2x 30 min/w)?

Response: The amount of regular training (i.e., soccer-specific training of 360–420 minutes per week and athletic training of 90–180 minutes per week) was equally distributed between groups. Consequently, the detected differences in static and dynamic balance outcomes (see Results) are related to the specificity of the respective training modality (i.e., unilateral singlemode BT group vs. unilateral combined BT+PT group vs. active control group).

Reply: I do not think my concern was fully addressed. My point was less about equal distribution between groups, but rather about the interpretability of relatively small intervention effects in the context of a very high overall training volume. Even if background training was comparable between groups, it still remains difficult to isolate the contribution of the additional intervention. I still feel this is a considerable limitation.

Comment: L176: Six seconds seems to be rather short? Could you provide reliability data for this test?

Response: We agree with the reviewer that a 6-s measurement duration for the dynamic balance test condition seems to be rather short but are in agreement with previous studies [19,20] and was designed to minimize fatigue. However, reliability data obtained from the three datacollection trials (at pretesting) of the present study revealed the following findings (cf. Material and Methods section, Assessment of balance performance): “Reliability data were calculated from the present study and yielded for the assessment of static balance ICC values (95% CI) of 0.50 (0.34, 0.65) for ap-COP RMS (dominant leg), 0.60 (0.46, 0.73) for ap-COP RMS (nondominant leg), 0.55 (0.40, 0.68) for ml-COP RMS (dominant leg), and 0.60 (0.45, 0.72) for mlCOP RMS (non-dominant leg), indicating “fair to good” reliability [21]. For dynamic balance, ICC values (95% CI) amounted to 0.50 (0.35, 0.65) for ap-COP RMS (dominant leg), 0.47 (0.31, 0.62) for ap-COP RMS (non-dominant leg), 0.41 (0.24, 0.58) for ml-COP RMS (dominant leg), and 0.52 (0.36, 0.66) for ml-COP RMS (non-dominant leg), also indicating “fair to good” reliability.”

Reply: Thank you for adding the reliability analyses. However, I would argue that ICC values in the range of 0.4 to 0.6 are only moderate at best, particularly given the complexity of the subsequent interaction analyses. This should, in my opinion, be discussed more critically. Moreover, low ICC values inherently imply a substantial measurement error. For example, an ICC of 0.50 corresponds roughly to a SEM of 0.7 SD and an MDC95 of almost 2 SD. Thus, the relatively small observed differences and especially complex interaction effects should be interpreted considerably more cautiously.

Comment: L252: While I understand the argument for restricting the analysis for the success rate to levels 5-6 and the CoP to levels 3-4, this seems to be a post-hoc decision based on the distribution of your data, rather than a priori decision. Moreover, this results in different datasets for different outcomes and makes it more difficult to compare between the different outcomes.

Response: We agree with the reviewer that using levels 5 and 6 for the parameters success rate and levels 3 and 4 for the CoP-based parameters was a post-hoc decision rather than a priori decision. However, this was not based on the distribution of our data, rather than to avoid ceiling effects of levels 3 and 4 for the parameter success rate and to avoid bias due to selective drop-out (floor effects) of level-5 and level-6 trials from less-balanced players for CoP-based parameters (cf. Results, Preprocessing and overview). This resulted in different datasets for different outcomes. Consequently, we did not compare our findings between the different outcomes but used a three-step analysis (first step: binary parameters; second step: CoP-based indices; third step: LSI indices). The discussion of the results also follows this structure. When drawing conclusions, we believe that the results of the more sophisticated, in-depth analysis (i.e., CoP-based indices) are particularly relevant. We have therefore focused on these results in formulating our conclusions.

(cf. Abstract): “Given the greater improvements in parameters obtained from the more in-depth analyses (i.e., CoP-based indices) in favor of the combined BT+PT, this training modality seems to be more suitable for enhancing interlimb postural control.“

(cf. Conclusions): “The greater effectiveness of combined BT+PT in parameters resulting from more sophisticated, in-depth analyses suggest that this training modality is probably better suited to positively influencing interlimb postural control.“

Reply: I appreciate the transparent acknowledgment that this was a post-hoc decision. However, I still consider this problematic. Even if motivated by floor- and ceiling-effects, the approach still results in outcome-specific selection of data subsets after inspection of the data. Also, the subsequent prioritization of the CoP-based analyses appears partly post-hoc as well. I still feel that this complicates the interpretation of the findings.

Comment: L228: The model includes multiple main effects and several interaction terms (including 3-way interactions) with a comparably large sample size for sport science papers, but still relatively small sample size of n = 57). This seems quite complex and raises the concern of the models being overfitted. Are all these interactions theoretically justified? This is especially relevant as some findings are based on bivariate classification of results into significant/non-significant around p-values close to the traditional 0.05-threshold in complex interaction terms. More importantly, many of the key conclusions rely on higherorder interactions. For example, effects such as time x group x condition x difficulty level x leg (implicitly decomposed across multiple 3-way terms) are very difficult to interpret. From a physiological perspective, I’m not convinced that such interaction structures are meaningful? This should at least be discussed critically. Moreover, you analyze multiple dependent variables, sometimes after computing the dependent variable as a ratio. I feel that it would strengthen the paper if there would be a clearly defined primary outcome.

Response: We agree with the reviewer that the experiment implemented a complex design. The three-factor interactions were expected for observed values (i.e., the basic group x time interaction for any intervention design was expected to be moderated by at least one of the other design factors). Therefore, we opted to specify the expected effects as a priori contrasts (rather than following up significant omnibus F-tests with multiple comparisons; see Hays [16] and Schad et al. [15]. For clarification, we have added the following information to the Material and Methods section (cf. Inferential statistics): “We estimated fixed effects for the two contrasts defined for control and training groups, pretest/posttest, static/dynamic balance condition, task difficulty level, and dominant/non- dominant leg. The first contrast (C1) tested the average of the two training groups against the active CON group; the second contrast (C2) tested the difference between the two training groups. Contrasts with effect coding were used for the other factors. We also included all simple interactions between these main effects and six theoretically relevant three-factor interactions arising from the combination of time × (C1 | C2) × (balance condition | task difficulty level leg). These contrasts and associated interactions were dictated by the design and specified a priori with a clear expectation about the profile of means, not as omnibus F-tests to be followed up with post-hoc t-tests adjusted for multiple comparisons [15,16]. The design was used both for the GLMM for the parameter success rate and the LMM for the CoP-based parameters. For unbalanced designs, (G)LMMs preserve statistical power better than repeated-measures ANOVAs.”

Reply: I appreciate the additional clarification regarding the contrast structure and the rationale for the analyses. However, I still remain unconvinced regarding the overall complexity of the statistical models. I understand the argument that the contrasts were specified a priori and dictated by the experimental design. However, this does not fully address the core issue, whether the resulting higher-order interaction structures are physiologically meaningful and interpretable. In particular, many of the central conclusions rely on complex interactions involving combinations of time, intervention, condition, task difficulty, and leg. From a physiological perspective, I still find it difficult to understand why the effects of the intervention should systematically depend on such complex combinations of factors. Moreover, I still feel that the manuscript insufficiently addresses the issue of multiple hypotheses. While the contrasts may have been planned a priori (as written above without a clear physiological explanation for the complex interactions), the manuscript nevertheless tests a large number of interaction effects across multiple dependent variables and partially non-overlapping datasets. This is especially relevant given that several findings are close to the conventional p = 0.05 threshold and the low reliability (see above for the ICC comment), which will lead to interaction estimates becoming unstable. Thus, I still feel that the current analysis substantially increases the likelihood of spurious and non-replicable findings. This is still a major limitation and should be clearly labeled as a limitation.

Reviewer #3: General comments

This is a nicely written manuscript. The rationale is justified, and the outcomes would add to the existing literature. I particularly find the statistical analysis quite sophisticated. However, there are a few concerns and suggestions that I would like to invite the authors to take into account.

Specific comments

Abstract

Keywords seem to be missing

The objective is unclear. What do you mean by "of both training modalities"? The training modalities should be explicitly mentioned; otherwise, this leaves the reader unsure about the independent variables

Last statement: This statement reads awkwardly, particularly toward its end

Introduction

The rationale is sound. For some reason, part of the content does not exist. Although the study can be classified under incremental science, I find the outcomes, practically speaking, quite interesting.

Line 63 For some reason, the rest of the text is gone and is not visible.

Methods

Please use body mass instead of weight.

Line 264 This is partly inconsistent and redundant with what you mentioned at the beginning of this subsection

Line 295 I would explicitly indicate that the duration of training was matched between the two experimental groups

Line 317 No parameters of internal training load were used. This could be added to the limitations.

Line 333 What about those who use both sides equally?

Line 344 Is this test-retest reliability, with, for instance, a couple of days in between, or between-trial (reps) reliability? Additionally, I don't think an ICC of 0.50 indicates good reliability; at best, it is moderate. Also, the reference (35) used is quite old. You could refer to https://www.sciencedirect.com/science/article/pii/S1556370716000158. Sorry if I missed it, did participants get familiarised with the tasks?

Line 348 Here, the reliability ranges from poor to moderate according to https://www.sciencedirect.com/science/article/pii/S1556370716000158. This could be added to the limitations as we can't be confident enough that the effects are due to training and not to poor reliability

Results

The statistical analysis and data interpretation are nicely detailed. The statistical approach used is quite sophisticated

Discussion

I prefer to specify that this is plyometric jump training (PJT). You could use this wording throughout the manuscript instead of the existing

Line 576 I suggest consistently specifying the sex of participants

Line 675 I would also add the poor to moderate reliability of the different parameters as a serious potential source of bias and, therefore, a limitation of this study that may affect the accuracy/veracity of findings. Additionally, the lack of injury monitoring and the potential associated with LSI could be added to the limitations. What about adding the main research perspectives, or follow-up studies that could be conducted?

**********

what does this mean?). If published, this will include your full peer review and any attached files.

If you choose “no”, your identity will remain anonymous but your review may still be made public.

**Do you want your identity to be public for this peer review?**  For information about this choice, including consent withdrawal, please see our Privacy Policy

Reviewer #2: No

Reviewer #3: **Yes:** Helmi Chaabene

**********

[NOTE: If reviewer comments were submitted as an attachment file, they will be attached to this email and accessible via the submission site. Please log into your account, locate the manuscript record, and check for the action link "View Attachments". If this link does not appear, there are no attachment files.]

To ensure your figures meet our technical requirements, please review our figure guidelines: https://journals.plos.org/plosone/s/figures

You may also use PLOS’s free figure tool, NAAS, to help you prepare publication quality figures: https://journals.plos.org/plosone/s/figures#loc-tools-for-figure-preparation.

NAAS will assess whether your figures meet our technical requirements by comparing each figure against our figure specifications.

# Author comment: Author response to Decision Letter 2

**DOI:** 10.1371/journal.pone.0358565.r004

10 Aug 2026

Manuscript Number: PONE-D-26-01855R1

Title: Effects of unilateral single-mode balance training compared to combined balance and plyometric training on soccer players’ interlimb asymmetry in static and dynamic balance performance

Responses to Reviewer #2:

COMMENT: I highly appreciate your revised manuscript! Although the manuscript improved, I have some further comments regarding the responses to my initial points. Please see specific comments below:

Response: We thank the reviewer for this affirmative comment. Further, we are grateful for the time and effort invested in providing constructive criticism. Below you will find the responses to your comments. All changes made in response to your comments were highlighted in grey in the revised manuscript.

COMMENT on the Abstract – The conclusion reads that the combined BT+PT is “presumably more suitable”. However, the results show that BT is superior for success rate (although they performed significantly worse during pre), BT+PT is superior for CoP, and that there are no differences for LSI. Thus, this does not seem fully consistent with the conclusion. Therefore, I would recommend toning this down and presenting a more balanced interpretation.

Response: We agree with the reviewer. We think the results of the in-depth analyses are particularly relevant. These are CoP-based indices that provide insight into the regulatory processes underlying balance performance. We have therefore changed the statement as follows: ”Given the greater improvements in parameters obtained from the more in-depth analyses (i.e., CoP-based indices) in favor of the combined BT+PT, this training modality seems to be more suitable for enhancing interlimb postural control.“

Reply: Even though this is now clearer and toned down in wording, I still feel that the manuscript selectively prioritizes the CoP-based outcomes over the other outcome domains. While I agree that CoP-based measures may provide a more detailed assessment of postural regulation, this does not necessarily make them more meaningful or more valid than the other outcomes. Importantly, superiority of BT+PT is still mainly concluded based on the outcome domain favoring this intervention, whereas the binary outcome favored BT and LSI outcomes showed largely no differences. Thus, I would still recommend presenting the findings in a more balanced manner and emphasizing the inconsistency across outcome domains more explicitly.

Response: We agree with the reviewer and changed the statement as follows: ”Due to the inconsistency across outcome domains, it cannot be concluded that one training mode is superior to the other. However, the more in-depth analysis (i.e., CoP-based indices) yields findings in favor of the combined BT+PT and suggests that regulatory processes, in particular, benefit from this training modality.“

COMMENT on L152: Participants were exhibited to a high amount of regular training (soccer + athletic + PE), which might also have included elements comparable to the additional intervention. Thus, it might be hard to isolate the contribution of the relatively low amount of additional training of the intervention (2x 30 min/w)?

Response: The amount of regular training (i.e., soccer-specific training of 360–420 minutes per week and athletic training of 90–180 minutes per week) was equally distributed between groups. Consequently, the detected differences in static and dynamic balance outcomes (see Results) are related to the specificity of the respective training modality (i.e., unilateral single-mode BT group vs. unilateral combined BT+PT group vs. active control group).

Reply: I do not think my concern was fully addressed. My point was less about equal distribution between groups, but rather about the interpretability of relatively small intervention effects in the context of a very high overall training volume. Even if background training was comparable between groups, it still remains difficult to isolate the contribution of the additional intervention. I still feel this is a considerable limitation.

Response: We agree with the reviewer and have added this aspect to the limitations paragraph (cf. Discussion): “Eighth, although the total training volume was distributed equally among the groups, the proportion of the additional intervention (i.e., 60 minutes per week) was smaller than the proportion of soccer-specific training (360–420 minutes per week) and athletic training (90–180 minutes per week). Therefore, it cannot be ruled out that the observed effects were caused solely by the intervention.”

COMMENT on L176: Six seconds seems to be rather short? Could you provide reliability data for this test?

Response: We agree with the reviewer that a 6-s measurement duration for the dynamic balance test condition seems to be rather short but it is in agreement with previous studies [19,20] and was designed to minimize fatigue. However, reliability data obtained from the three data-collection trials (at pretesting) of the present study revealed the following findings (cf. Material and Methods section, Assessment of balance performance): “Reliability data were calculated from the present study and yielded for the assessment of static balance ICC values (95% CI) of 0.50 (0.34, 0.65) for ap-COP RMS (dominant leg), 0.60 (0.46, 0.73) for ap-COP RMS (nondominant leg), 0.55 (0.40, 0.68) for ml-COP RMS (dominant leg), and 0.60 (0.45, 0.72) for mlCOP RMS (non-dominant leg), indicating “fair to good” reliability [21]. For dynamic balance, ICC values (95% CI) amounted to 0.50 (0.35, 0.65) for ap-COP RMS (dominant leg), 0.47 (0.31, 0.62) for ap-COP RMS (non-dominant leg), 0.41 (0.24, 0.58) for ml-COP RMS (dominant leg), and 0.52 (0.36, 0.66) for ml-COP RMS (non-dominant leg), also indicating “fair to good” reliability.”

Reply: Thank you for adding the reliability analyses. However, I would argue that ICC values in the range of 0.4 to 0.6 are only moderate at best, particularly given the complexity of the subsequent interaction analyses. This should, in my opinion, be discussed more critically. Moreover, low ICC values inherently imply a substantial measurement error. For example, an ICC of 0.50 corresponds roughly to a SEM of 0.7 SD and an MDC95 of almost 2 SD. Thus, the relatively small observed differences and especially complex interaction effects should be interpreted considerably more cautiously.

Response: The other reviewer also raised this issue. Consequently, we have changed “fair to good” to “poor to moderate” reliability. In addition, we have added this aspect to the limitations paragraph (cf. Discussion): “Sixth, between-trial test-retest reliability was poor to moderate for the dynamic balance task. Therefore, it cannot be ruled out that the low repeatability of data collection may have affected the accuracy of the present findings.”

COMMENT on L252: While I understand the argument for restricting the analysis for the success rate to levels 5-6 and the CoP to levels 3-4, this seems to be a post-hoc decision based on the distribution of your data, rather than a priori decision. Moreover, this results in different datasets for different outcomes and makes it more difficult to compare between the different outcomes.

Response: We agree with the reviewer that using levels 5 and 6 for the parameters success rate and levels 3 and 4 for the CoP-based parameters was a post-hoc decision rather than a priori decision. However, this was not based on the distribution of our data, rather than to avoid ceiling effects of levels 3 and 4 for the parameter success rate and to avoid bias due to selective drop-out (floor effects) of level-5 and level-6 trials from less-balanced players for CoP-based parameters (cf. Results, Preprocessing and overview). This resulted in different datasets for different outcomes. Consequently, we did not compare our findings between the different outcomes but used a three-step analysis (first step: binary parameters; second step: CoP-based indices; third step: LSI indices). The discussion of the results also follows this structure. When drawing conclusions, we believe that the results of the more sophisticated, in-depth analysis (i.e., CoP-based indices) are particularly relevant. We have therefore focused on these results in formulating our conclusions.

(cf. Abstract): „Given the greater improvements in parameters obtained from the more in-depth analyses (i.e., CoP-based indices) in favor of the combined BT+PT, this training modality seems to be more suitable for enhancing interlimb postural control.“

(cf. Conclusions): „The greater effectiveness of combined BT+PT in parameters resulting from more sophisticated, in-depth analyses suggest that this training modality is probably better suited to positively influencing interlimb postural control.“

Reply: I appreciate the transparent acknowledgment that this was a post-hoc decision. However, I still consider this problematic. Even if motivated by floor- and ceiling-effects, the approach still results in outcome-specific selection of data subsets after inspection of the data. Also, the subsequent prioritization of the CoP-based analyses appears partly post-hoc as well. I still feel that this complicates the interpretation of the findings.

Response: Thank you for highlighting this aspect again. Consequently, we emphasized the inconsistency across outcome domains more explicitly in the Abstract and Conclusions.

Abstract: ”Due to the floor and ceiling effects across outcome domains, it cannot be concluded that one training mode is superior to the other. However, the more in-depth analysis (i.e., CoP-based indices) yields findings in favor of the combined BT+PT and suggests that regulatory processes, in particular, benefit from this training modality.“

Conclusions: ”The floor and ceiling effects across outcome domains prevent definitive conclusion regarding the superiority of either training modality. However, the detailed analyses based on CoP-derived indices favor the combined BT+PT approach, suggesting that this training modality is particularly effective in enhancing balance regulatory processes.“

COMMENT on L228: The model includes multiple main effects and several interaction terms (including 3-way interactions) with a comparably large sample size for sport science papers, but still relatively small sample size of n = 57). This seems quite complex and raises the concern of the models being overfitted. Are all these interactions theoretically justified? This is especially relevant as some findings are based on bivariate classification of results into significant/non-significant around p-values close to the traditional 0.05-threshold in complex interaction terms. More importantly, many of the key conclusions rely on higher order interactions. For example, effects such as time x group x condition x difficulty level x leg (implicitly decomposed across multiple 3-way terms) are very difficult to interpret. From a physiological perspective, I’m not convinced that such interaction structures are meaningful? This should at least be discussed critically. Moreover, you analyze multiple dependent variables, sometimes after computing the dependent variable as a ratio. I feel that it would strengthen the paper if there would be a clearly defined primary outcome.

Response: We agree with the reviewer that the experiment implemented a complex design. The three-factor interactions were expected for observed values (i.e., the basic group x time interaction for any intervention design was expected to be moderated by at least one of the other design factors). Therefore, we opted to specify the expected effects as a priori contrasts (rather than following up significant omnibus F-tests with multiple comparisons; see Hays [16] and Schad et al. [15]. For clarification, we have added the following information to the Material and Methods section (cf. Inferential statistics): “We estimated fixed effects for the two contrasts defined for control and training groups, pretest/posttest, static/dynamic balance condition, task difficulty level, and dominant/non- dominant leg. The first contrast (C1) tested the average of the two training groups against the active CON group; the second contrast (C2) tested the difference between the two training groups. Contrasts with effect coding were used for the other factors. We also included all simple interactions between these main effects and six theoretically relevant three-factor interactions arising from the combination of time × (C1 | C2) × (balance condition | task difficulty level leg). These contrasts and associated interactions were dictated by the design and specified a priori with a clear expectation about the profile of means, not as omnibus F-tests to be followed up with post-hoc t-tests adjusted for multiple comparisons [15,16]. The design was used both for the GLMM for the parameter success rate and the LMM for the CoP-based parameters. For unbalanced designs, (G)LMMs preserve statistical power better than repeated-measures ANOVAs.”

Reply: I appreciate the additional clarification regarding the contrast structure and the rationale for the analyses. However, I still remain unconvinced regarding the overall complexity of the statistical models. I understand the argument that the contrasts were specified a priori and dictated by the experimental design. However, this does not fully address the core issue, whether the resulting higher-order interaction structures are physiologically meaningful and interpretable. In particular, many of the central conclusions rely on complex interactions involving combinations of time, intervention, condition, task difficulty, and leg. From a physiological perspective, I still find it difficult to understand why the effects of the intervention should systematically depend on such complex combinations of factors. Moreover, I still feel that the manuscript insufficiently addresses the issue of multiple hypotheses. While the contrasts may have been planned a priori (as written above without a clear physiological explanation for the complex interactions), the manuscript nevertheless tests a large number of interaction effects across multiple dependent variables and partially non-overlapping datasets. This is especially relevant given that several findings are close to the conventional p = 0.05 threshold and the low reliability (see above for the ICC comment), which will lead to interaction estimates becoming unstable. Thus, I still feel that the current analysis substantially increases the likelihood of spurious and non-replicable findings. This is still a major limitation and should be clearly labeled as a limitation.

Response: We agree with the reviewer that this is a complex experiment with limitations. We have presented a transparent analysis, acknowledged the limitations, and did not want to overstate conclusions. We cannot expect agreement with all our decisions but think that our analyses fall in the space where reasonable people should be allowed to disagree. We will certainly make data and scripts available for alternative analyses.

We comment on two sentences in your comment:

The reviewer writes “From a physiological perspective, I still find it difficult to understand why the effects of the intervention should systematically depend on such complex combinations of factors.” The dependency is a necessary consequence of any experimental design with pretest/posttest, control and experimental conditions and theoretically motivated additional factors. One alternative is to analyze change scores (i.e., remove the pretest/posttest factor from the interactions). Another conceptually, but not statistically simpler solution is to analyze posttest scores and include pretest as a covariate. These alternatives have their own weaknesses well documented in the literature.

The reviewer also writes: “Thus, I still feel the current analysis substantially increases the likelihood of spurious and non-replicable findings. This is still a major limitation and should be clearly labeled as a limitation.” We are not in disagreement with this sentiment. In our opinion, the most promising way forward is replication. We think that our experiment and analyses provide a necessary and suitable platform for such a pe

Attachment Submitted filename: response_to_reviewers_auresp_2.docx

# Peer review: Decision Letter 2

**DOI:** 10.1371/journal.pone.0358565.r005

**Authors:** Eduard Kurz

3 Sep 2026

Effects of unilateral single-mode balance training compared to combined balance and plyometric training on soccer players’ interlimb asymmetry in static and dynamic balance performance

PONE-D-26-01855R2

Dear Dr. Muehlbauer,

We’re pleased to inform you that your manuscript has been judged scientifically suitable for publication and will be formally accepted for publication once it meets all outstanding technical requirements.

Within one week, you’ll receive an e-mail detailing the required amendments. When these have been addressed, you’ll receive a formal acceptance letter and your manuscript will be scheduled for publication.

An invoice will be generated when your article is formally accepted. Please note, if your institution has a publishing partnership with PLOS and your article meets the relevant criteria, all or part of your publication costs will be covered. Please make sure your user information is up-to-date by logging into Editorial Manager at Editorial Manager® and clicking the ‘Update My Information' link at the top of the page. For questions related to billing, please contact billing support.

If your institution or institutions have a press office, please notify them about your upcoming paper to help maximize its impact. If they’ll be preparing press materials, please inform our press team as soon as possible -- no later than 48 hours after receiving the formal acceptance. Your manuscript will remain under strict press embargo until 2 pm Eastern Time on the date of publication. For more information, please contact onepress@plos.org.

Kind regards,

Eduard Kurz, PhD

Academic Editor

PLOS One

Additional Editor Comments (optional):

Dear Prof. Muehlbauer,

I am writing to inform you that your manuscript "Effects of unilateral single-mode balance training compared to combined balance and plyometric training on soccer players’ interlimb asymmetry in static and dynamic balance performance" has been accepted for publication in PLOS One.

With best regards,

Eduard Kurz, PhD

Reviewers' comments:

Reviewer's Responses to Questions

**Comments to the Author**

Reviewer #2: (No Response)

Reviewer #4: All comments have been addressed

**********

2. Is the manuscript technically sound, and do the data support the conclusions?

Reviewer #2: Yes

Reviewer #4: Yes

**********

3. Has the statistical analysis been performed appropriately and rigorously?

Reviewer #2: Yes

Reviewer #4: Yes

**********

4. Have the authors made all data underlying the findings in their manuscript fully available?

The PLOS Data policy

Reviewer #2: Yes

Reviewer #4: Yes

**********

5. Is the manuscript presented in an intelligible fashion and written in standard English?

Reviewer #2: Yes

Reviewer #4: Yes

**********

Reviewer #2: Dear editorial board, dear authors,

I am delighted to have the opportunity of reviewing this revised version of the manuscript. I commend the authors for the effort put into this second revision: the balanced interpretation in the Abstract and Conclusions, the reliability data and their re-labelling, the disclosure of how the difficulty levels were selected, and the two new limitations addressing training volume and model complexity together deal with the points I raised in the previous round. In particular, I appreciate the transparency of the added statement that the choice of measures was informed by the outcomes. As far as I’m concerned, in its current form this manuscript is suited for publication. I have only some minor suggestions, one of which concerns a sentence that in my reading says the opposite of what is meant.

L558-560: “Therefore, it cannot be ruled out that the observed effects were caused solely by the intervention.” I think this sentence is inverted. Given the preceding sentence (60 min/week of intervention against 360-420 min/week soccer-specific and 90-180 min/week athletic training), the intended meaning must be that it cannot be assumed that the observed effects were caused solely by the intervention, or that a contribution of the concurrent regular training cannot be ruled out.

L560-562: The findings are described here as “based on partially overlapping data”, whereas the Methods state that success rate and CoP measures were analysed on “two non-overlapping subsets of the observations” (L290-291), and the Results confirm that levels 5-6 and levels 3-4 are disjoint (L313-317). Only a minor point, but the two passages should be aligned.

Reviewer #4: Thanks for revising your work. The revision has really boosted the quality and relevance of the study, and I have no further comments.

**********

what does this mean?). If published, this will include your full peer review and any attached files.

If you choose “no”, your identity will remain anonymous but your review may still be made public.

**Do you want your identity to be public for this peer review?**  For information about this choice, including consent withdrawal, please see our Privacy Policy

Reviewer #2: No

Reviewer #4: No

**********

# Editor report: Acceptance letter

**DOI:** 10.1371/journal.pone.0358565.r006

**Authors:** Eduard Kurz

PONE-D-26-01855R2

PLOS One

Dear Dr. Muehlbauer,

I'm pleased to inform you that your manuscript has been deemed suitable for publication in PLOS One. Congratulations! Your manuscript is now being handed over to our production team.

At this stage, our production department will prepare your paper for publication. This includes ensuring the following:

* All references, tables, and figures are properly cited

* All relevant supporting information is included in the manuscript submission,

* There are no issues that prevent the paper from being properly typeset

You will receive further instructions from the production team, including instructions on how to review your proof when it is ready. Please keep in mind that we are working through a large volume of accepted articles, so please give us a few days to review your paper and let you know the next and final steps.

Lastly, if your institution or institutions have a press office, please let them know about your upcoming paper now to help maximize its impact. If they'll be preparing press materials, please inform our press team within the next 48 hours. Your manuscript will remain under strict press embargo until 2 pm Eastern Time on the date of publication. For more information, please contact onepress@plos.org.

You will receive an invoice from PLOS for your publication fee after your manuscript has reached the completed accept phase. If you receive an email requesting payment before acceptance or for any other service, this may be a phishing scheme. Learn how to identify phishing emails and protect your accounts at https://explore.plos.org/phishing.

If we can help with anything else, please email us at customercare@plos.org.

Thank you for submitting your work to PLOS One and supporting open access.

Kind regards,

PLOS One Editorial Office Staff

on behalf of

Dr. Eduard Kurz

Academic Editor

PLOS One
